# Hypoxia-enhanced YAP1-EIF4A3 interaction drives circ_0007386 circularization by competing with *CRIM1* pre-mRNA linear splicing and promotes non-small cell lung cancer progression

**DOI:** 10.1186/s13046-024-03116-6

**Published:** 2024-07-20

**Authors:** Lixia Li, Dewei Liu, Tingting Chen, Chunhui Wei, Youping Qiao, Weiliang Liu, Yanmei Liang, Zhu Liang, Chunyuan Chen, Dongming Li, Bin Wu, Xuanna Zhao, Dan Huang, Dong Wu

**Affiliations:** 1 Department of Respiratory and Critical Care Medicine, Affiliated Hospital of Guangdong Medical University, Zhanjiang, 524000 China; 2 Cancer Hospital, Affiliated Hospital of Guangdong Medical University, Zhanjiang, China; 3 Department of Cardiothoracic Surgery, Affiliated Hospital of Guangdong Medical University, Zhanjiang, China

**Keywords:** Non-small cell lung cancer, Circ_0007386, miR-383-5p, CIRBP, EIF4A3, YAP1, Apoptosis, Proliferation, Hypoxia

## Abstract

**Background:**

The progression of non-small cell lung cancer (NSCLC) is significantly influenced by circular RNAs (circRNAs), especially in tumor hypoxia microenvironment. However, the precise functions and underlying mechanisms of dysregulated circRNAs in NSCLC remain largely unexplored.

**Methods:**

Differentially expressed circRNAs in NSCLC tissues were identified through high-throughput RNA sequencing. The characteristics of circ_0007386 were rigorously confirmed via Sanger sequencing, RNase R treatment and actinomycin D treatment. The effects of circ_0007386 on proliferation and apoptosis were investigated using CCK8, cloning formation assays, TUNEL staining, and flow cytometry assays in vitro. In vivo, xenograft tumor models were used to evaluate its impact on proliferation. Mechanistically, the regulatory relationships of circ_0007386, miR-383-5p and CIRBP were examined through dual luciferase reporter assays and rescue experiments. Additionally, we detected the binding of EIF4A3 to *CRIM1* pre-mRNA by RNA immunoprecipitation and the interaction between YAP1 and EIF4A3 under hypoxic conditions by co-immunoprecipitation.

**Results:**

Our investigation revealed a novel circRNA, designated as circ_0007386, that was upregulated in NSCLC tissues and cell lines. Circ_0007386 modulated proliferation and apoptosis in NSCLC both in vitro and in vivo. Functionally, circ_0007386 acted as a sponge for miR-383-5p, targeting CIRBP, which influenced NSCLC cell proliferation and apoptosis via the PI3K/AKT signaling pathway. Furthermore, under hypoxic conditions, the interaction between YAP1 and EIF4A3 was enhanced, leading to the displacement of EIF4A4 from binding to *CRIM1* pre-mRNA. This facilitated the back-splicing of *CRIM1* pre-mRNA, increasing the formation of circ_0007386. The circ_0007386/miR-383-5p/CIRBP axis was significantly associated with the clinical features and prognosis of NSCLC patients.

**Conclusions:**

Circ_0007386, regulated by YAP1-EIF4A3 interaction under hypoxia conditions, plays an oncogenic role in NSCLC progression via the miR-383-5p/CIRBP axis.

**Supplementary Information:**

The online version contains supplementary material available at 10.1186/s13046-024-03116-6.

## Introduction

Lung cancer remains the primary cause of cancer-related mortality globally, with non-small cell lung cancer (NSCLC) accounting for 85% of these cases [1]. The processes of proliferation and apoptosis are pivotal in the development and progression of lung cancer [2]. The Cold-inducible RNA binding protein (CIRBP), a member of the glycine-rich RNA binding protein family, plays a role in several cellular processes including the regulation of gene expression, cell cycle control, and apoptosis in both inflammation and cancer contexts [3, 4]. In bladder cancer, CIRBP promotes oncogenesis by activating the ERK1/2/p38 signaling pathway, enhancing cell proliferation and migration [5]. Conversely, in pancreatic cancer, CIRBP acts as a tumor suppressor, inhibiting cell growth via the p53/GPX4 pathway [6]. And in NSCLC, CIRBP activates Wnt/β-catenin signaling via CTNNB1 to increase proliferation and enhance invasion/metastasis [7]. However, the precise mechanisms by which CIRBP influences NSCLC apoptosis progression still needs further exploration.

Circular RNAs (circRNAs), which arise from the cyclization of a specific exon of a maternal gene, exhibit higher stability compared with linear mRNA [8]. These circRNAs have been identified as key players in human cancers, regulating target genes through various mechanisms, including acting as sponges for microRNAs (miRNAs) to fulfill critical biological roles [9]. miRNAs, a subclass of non-coding RNAs, bind to the 3’ untranslated region (UTR) of target mRNAs, affecting their translation inhibition or degradation. There is increasing evidence of circRNA dysregulation in tumor progression [10, 11]. Moreover, the recent discovery of hsa_circ_0007386 inducing apoptosis in arterial smooth muscle cells, thereby aggravating the progression of thoracic aortic dissection [12], highlights the significance of circRNAs. Nevertheless, the role of circ_0007386 in cancer progression remains underinvestigated.

Hypoxia, an essential microenvironmental factor, is known to exacerbate cancer progression and metastasis by facilitating angiogenesis, metastasis, resistance to apoptosis, and decreased sensitivity to chemotherapy and radiotherapy [13]. In our previous study, we explored the role of hypoxia-inducible factor-1α (HIF1α) in the biosynthesis of circ_0001875 [14] under hypoxia conditions, a focus of our research team’s ongoing efforts.

Several studies have reported that hypoxia stimulates the expression of Yes1 associated transcriptional regulator (YAP1), which is implicated in tumor progression [15, 16]. The eukaryotic translation initiation factor 4A3 (EIF4A3), a crucial component of the exon junction complex, plays a pivotal role in tumor regulation [17, 18]. Notably, EIF4A3 has been identified to act as an RNA-binding protein (RBP), capable of binding to *ASAP1* mRNA to facilitate the formation of circRNA ASAP1 [19]. The potential interaction between EIF4A3 and YAP1, particularly in the context of hypoxia-induced YAP1 involvement in circRNA production, remains to be elucidated.

This study aimed to elucidate the formation mechanism and the role of dysregulated circRNAs in NSCLC, with a specific focus on circ_0007386. Our investigations revealed the oncogenic potential of circ_0007386, demonstrating its ability to enhance NSCLC cell proliferation and suppress apoptosis both in vivo and in vitro. We also identified that circ_0007386 functions as a miR-383-5p sponge, modulating the impact of CIRBP on cell proliferation. Furthermore, we found that EIF4A3 inhibits the circularization of circ_0007386, a process that is hindered by YAP1’s competitive binding to EIF4A3. Importantly, the expression levels of circ_0007386, miR-383-5p, and CIRBP were strongly associated with the prognosis of NSCLC patients. Collectively, our findings underscore the significance of circ_0007386 as a diagnostic, therapeutic, and prognostic indicator for NSCLC.

## Methods

### Cell culture and transfection

NSCLC cell lines (A549, H838, H1299,H1975), human normal lung epithelial cells (BEAS-2B), and 293 A cells were sourced from the Chinese Academy of Sciences in Shanghai, China. These cells were cultured in DMEM medium (Gibco, Grand Island, NY, USA) supplemented with 10% fetal bovine serum (FBS) and incubated in a humidified atmosphere with 5% CO_2_ at 37 °C. For hypoxia experiments, the cells were cultured at 37 °C in a humidified hypoxic chamber (Forma Scientific, Marietta, OH, USA) with 1% O_2_ and 5% CO_2_.

Short interfering RNAs (siRNAs) targeting circ_0007386, CIRBP, YAP1, and EIF4A3, along with miRNA mimics, inhibitors, and their respective negative controls were obtained from GenePharma (Shanghai, China). Additionally, overexpression plasmids for circ_0007386, CIRBP, YAP1, and EIF4A3 were synthesized using their full-length cDNA sequences, also provided by GenePharma. For transient transfections, Lipofectamine RNAiMAX (Invitrogen, Carlsbad, CA, USA) was used for siRNAs and miRNA mimics/inhibitors, whereas Lipofectamine 3000 (Invitrogen) was employed for overexpression plasmids. A549 and H1299 cells underwent transfection with a circ_0007386 overexpression plasmid (pLCDH-circ_0007386), a knockdown vector (sh-circ_0007386), and a control vector (GenePharma). Stable cell lines were then generated through puromycin selection (Sigma-Aldrich, USA) for 2–3 weeks. The sequences for all constructs utilized in this study can be found in Table S1.

### Tissue samples

Between 2019 and 2022, 80 pairs of NSCLC and corresponding adjacent non-tumor tissues were procured from patients who underwent surgery at the Affiliated Hospital of Guangdong Medical University. Notably, these patients had not undergone any preoperative chemotherapy or radiotherapy. To maintain tissue integrity, immediate storage in liquid nitrogen was ensured. Ethical approval for this study was obtained from the Ethics Committee of the Affiliated Hospital of Guangdong Medical University, and informed consent was acquired from all participants through their voluntary signing of consent forms.

### Animal models

For this study, female nude mice (BALB/c) at 4 weeks of age were obtained from Guangdong Medical Experimental Animal Center. To establish subcutaneous tumor models, mice were injected with 6 × 10^6^ cells each, using transfected cells injected subcutaneously into the armpits. The mice were randomly assigned to groups for each experiment. In the first experiment, groups consisted of the control shRNA (sh-NC) and circ_0007386 shRNA (sh-circ_0007386), with six mice in each group. In the second experiment, the groups included the control vector (pLCDH vector) and the circ_0007386 overexpression vector (pLCD circ_0007386), also with six mice per group. Tumor growth was monitored and measured weekly using a Vernier scale, and tumor volume was calculated using the formula V = (width^2^ × length)/2. Following a four-week period of tumor growth, the mice were euthanized, and the subcutaneous tumors were harvested for histological analysis. The presence of cancer cells was confirmed by hematoxylin and eosin (HE) staining. Additionally, immunohistochemistry (IHC) staining was performed using antibodies against CIRBP (Proteintech, USA, 1:200), Ki67 (Servicebio, China, 1:200), BAX (Proteintech, USA, 1:2000) and Bcl2 (Servicebio, China, 1:500). Images were captured using an Olympus Corporation microscope.

The Animal Ethics Committee of Guangdong Medical University conducted a thorough review and granted approval for all animal experiments conducted in this study. The mice were housed in specific pathogen-free (SPF) conditions and received appropriate care at the Experimental Animal Center of the Affiliated Hospital of Guangdong Medical University.

### RNA-seq

We collected three pairs of NSCLC tissues and adjacent tissues from NSCLC patients who had undergone surgical resection in the Department of Cardiothoracic Surgery, Affiliated Hospital of Guangdong Medical University (Zhanjiang, China). No antitumor treatment was performed before the surgery. All samples were frozen in liquid nitrogen following surgical resection until analyses. Total RNA was isolated from three pairs of NSCLC tissues and adjacent normal tissues using TRIzol Reagent (Invitrogen, CA, USA). The quality of the RNA was assessed by an Agilent 2100 machine to ensure its integrity, and only samples with an RNA integrity number > 7.0 were used in the experiments. Ribosomal RNA was then removed from the samples using a RiboMinus Eukaryote kit (Qiagen, Valencia, CA, USA). The RNA-seq library was prepared and deep sequenced using an Illumina HiSeq 2000 instrument (Illumina, San Diego, CA, USA) to obtain paired-end reads. The sequencing data analysis was performed using the Edger software(v3.16.5).

### RNA extraction and quantitative real-time polymerase chain reaction (RT-qPCR)

To extract total RNA from tissues and cells, Trizol reagent (Invitrogen) was used following the manufacturer’s instructions. Subsequently, cDNA synthesis was carried out using Evo M-MLV RT Premix (AG, Hunan, China). RT-qPCR analysis was performed using either the ABI7500 system (Applied Biosystems, Foster City, CA, USA) or the LightCycler 480 system (Roche Applied Science). *U6* and *β-actin* mRNA served as internal loading controls for miRNA and mRNA analysis, respectively. The primers for this study (provided in Table S2) were procured from Sango Biotech (Shanghai, China). Each sample was analyzed in a minimum of three replicates, and data analysis was conducted using the 2^−ΔΔCT^ statistical method.

### RNase R treatment and actinomycin D (ActD) treatment

For RNase R treatment, cells were subjected to total RNA extraction (2 µg) and were then incubated at 37 °C for 15 min with RNase R (3 U/µg) supplied by Epicentre Technologies Corporation (Madison, WI, USA). In the case of ActD treatment, cells were incubated with ActD (2 µg/mL) obtained from Beyotime (Shanghai, China) for predetermined periods.

### Cell counting Kit-8 (CCK8) assay and cloning formation assay

In the CCK-8 assay (Beyotime), transfected cells were seeded in 96-well plates at a density of 2000 cells in 100 µL of medium per well. At predetermined intervals (0, 24, 48, 72, and 96 h) after plating, 10 µL of CCK-8 reagent was added to each well, followed by incubation at 37 °C for 1 h. Cell viability was assessed by measuring absorbance at 450 nm.

For the clone formation assay, post-transfection cells were seeded in 6-well plates at a density of 1000 cells per well and cultured at 37 °C for two weeks. Cells were then fixed with methanol for 30 min and stained with 0.1% crystal violet. Imaging and cell counting were performed to evaluate the results.

### Flow cytometry assay

The treated cells underwent incubation for 48 h, followed by elution and collection in a flow cytometry tube. As per the flow cytometry instructions, staining was conducted for 30 min using BD, USA reagents.

### TUNEL staining assay

The TUNEL assay was performed according to the protocol provided with the TUNEL Apoptosis Detection Kit (Beyotime, China). Cells were fixed in a 4% paraformaldehyde solution for 30 min, then permeabilized with PBS containing 0.3% Triton X-100. The TUNEL detection solution was applied and the mixture incubated in the dark at 37 °C for 60 min. Following the addition of DAPI staining solution, samples were examined and imaged using Olympus Laser confocal microscopy (Olympus Corporation, Tokyo, Japan).

### Western blot

Transfected cells were lysed using RIPA buffer (Beyotime) supplemented with PMSF. Protein concentration was then determined using BCA reagent (Beyotime). Equal amounts of proteins were subjected to electrophoresis on a 10% SDS-PAGE gel and then transferred onto a PVDF membrane (Millipore, Billerica, MA, USA). The membrane was blocked with 5% BSA for 1 h at room temperature before incubating overnight with the primary antibody at 4 °C. The membrane was subsequently treated with the corresponding HRP-labeled secondary antibody for 1 h. Protein signals were detected using BeyoECL star (Beyotime). A comprehensive list of all the antibodies employed in this study can be found in Table S3.

### Fluorescence in situ hybridization (FISH) and immunofluorescence staining

Cells were fixed in a confocal dish using a 4% paraformaldehyde solution for 30 min. Permeabilization was carried out using PBS containing 0.5% Triton X-100. For FISH, cells incubated overnight at 37 °C with a cy3-labeled circ_0007386 or miR-383-5p probe supplied by GenePharma. Afterward, cells were treated with anti-fade mounting medium and DAPI staining. Olympus Laser confocal microscopy (Olympus Corporation, Tokyo, Japan) was used to capture the signals.

### Nuclear cytoplasm separation experiment

An extraction Kit (Invitrogen) was used to perform cytoplasmic and nuclear RNA isolation. Then, the RNA expression levels of circ_0007386, as well as β-actin (a cytoplasmic protein marker) and U6 (a nuclear protein marker), were examined in the two fractions.

### RNA-binding protein immunoprecipitation (RIP) assay

The RIP assays were conducted using the Magna RIP RNA-Binding Protein Immunoprecipitation Kit (Millipore). Roughly 2 × 10^7^ cells were lysed in RIP lysis buffer containing protease and RNase inhibitors. Cell lysates were then incubated with either IgG or anti-EIF4A3 antibody-conjugated magnetic beads (Millipore) and rotated at 4 °C overnight. The RNA/bead complexes were washed with RIP wash buffer, reconstituted in Proteinase K buffer to purify the immunoprecipitated RNA, which was subsequently reverse transcribed into cDNA for RT-qPCR analysis.

### Dual luciferase reporter assay

Luciferase reporter plasmids for circ_0007386 and CIRBP, including both wild-type and mutant-type constructs, were synthesized by GenePharma. Cells were seeded in 24-well plates and grown until reaching approximately 60% confluence. Subsequently, cells were transfected with luciferase plasmids, the Renilla control plasmid, and miRNA mimics. The activity of Firefly and Renilla luciferase was measured using the Dual Luciferase Assay System (Promega), enabling the detection and quantification of luciferase activity.

### Co-immunoprecipitation (CoIP) assay

Transfected cells were lysed using RIPA buffer (Beyotime) and subsequently incubated overnight at 4 °C on a rotator with normal IgG or anti-EIF4A3 antibodies. Antibody-protein complexes were captured by incubating with Dynabeads-protein G beads (Beyotime) for 2 h. The complexes were then analyzed using western blot analysis.

### Bioinformatics analysis

CircBase provided the sequence data for circ_0007386. The use of Circinteractome (https://circinteractome.nia.nih.gov) facilitated the identification of target miRNAs for circ_0007386. Target genes were predicted using multiple platforms, including TargetScan (https://targetscan.org/), ENCORI (http://starbase.sysu.edu.cn/index.php), and miRDB (https://mirdb.org/). CircInteractome, CircFunBase (https://bis.zju.edu.cn/CircFunBase), and ENCORI were employed to predict the RBP regulating circ_0007386 formation.

### Statistical analysis

Statistical analysis was conducted using GraphPad Prism 8.0 and SPSS 23.0 software. Comparative analysis involved the use of either Student’s *t*-test (two-tailed) or one-way ANOVA, followed by Bonferroni’s post-hoc test for further assessment. Survival curves were generated using the Kaplan–Meier method. The relationships between circ_0007386, miR-383-5p, and CIRBP were evaluated using Pearson’s correlation test. A *p*-value < 0.05 was considered statistically significant.

## Results

### Circ_0007386 is upregulated in NSCLC cells and tissues

**Fig. 1:**
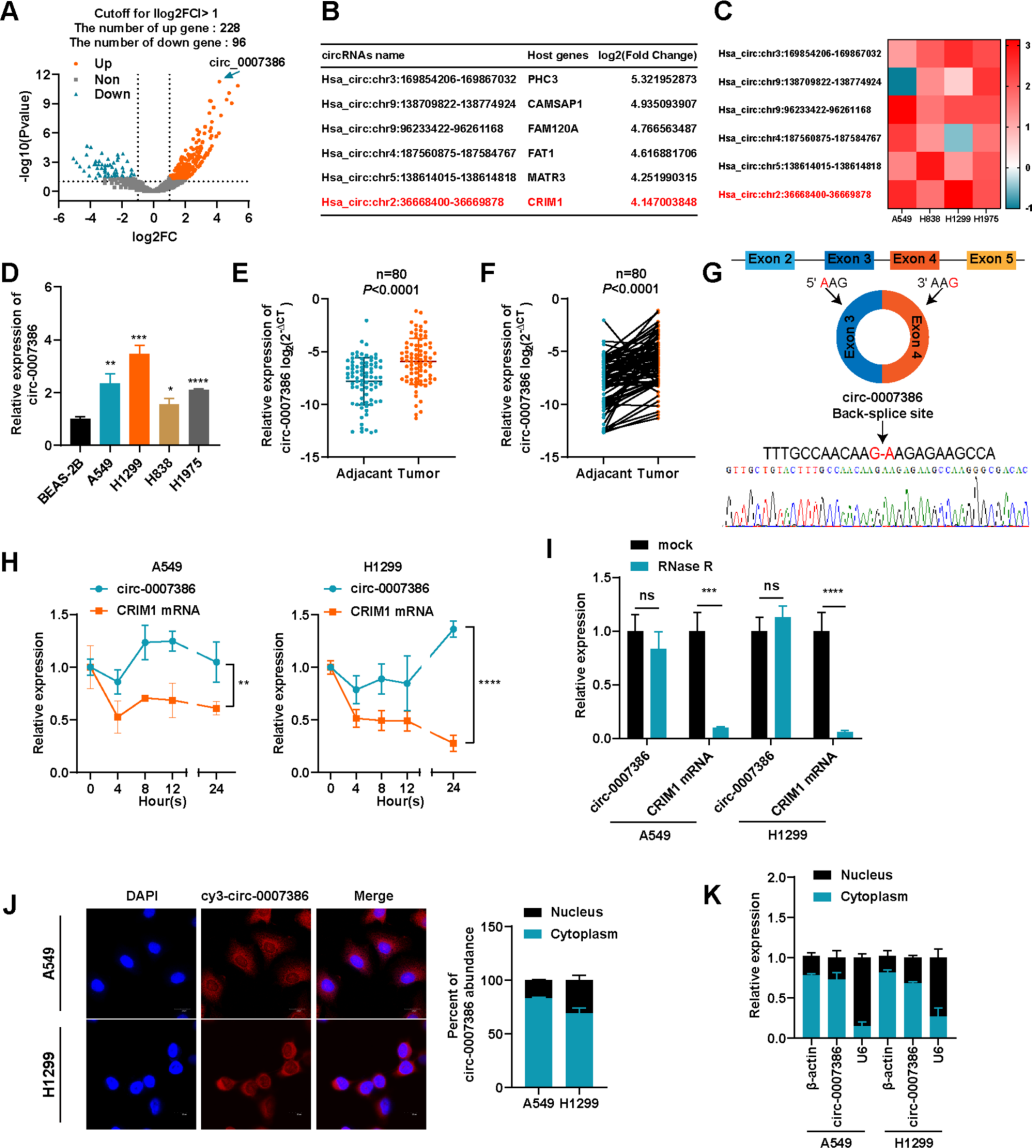
Expression and characterization of circ_0007386 in NSCLC cells and tissues. **(A)** Volcano plots showing the differentially expressed circRNAs in NSCLC tissue versus matched normal tissue. **(B)** A list of the top six dysregulated circRNAs. **(C)** RT-qPCR analysis was performed to compare the expression levels of the six most dysregulated circRNAs in A549, H838, H1299, and H1975 cells with their expression levels in BEAS-2B cells. **(D)** Relative expression levels of circ_0007386 in BEAS-2B cells and NSCLC cell lines were detected by RT-qPCR. **E-F.** RT-qPCR was conducted to assess the relative expression of circ_0007386 in NSCLC tissues and adjacent nontumor tissues (*n* = 80). **G.** A schematic of the genomic location and back splicing of circ_0007386 with the splicing site verified by Sanger sequencing. **H.** circ_0007386 and *CRIM1* mRNA expression were examined by RT-qPCR in NSCLC cells after actinomycin D treatment on 0 h, 4 h, 8 h, 12 h, 24 h respectively. **I.** circ_0007386 and *CRIM1* mRNA expression were examined in NSCLC cells after RNase R treatment. **J.** FISH assay showed that circ_0007386 (Cy3-labeled probe) was abundant in the cytoplasm, and DAPI was used to stain the nucleus. Images are shown at 600× magnification. Scale bar = 20 μm. **K.** The localization of circ_0007386 in A549 and H1299 cells detected by nuclear-cytoplasmic separation experiment. Data are shown as the means ± SD. *p < 0.05, **p < 0.01, ***p < 0.001, ****p < 0.0001

To investigate the involvement of circRNAs in NSCLC, a RNA-seq analysis was performed on three pairs of NSCLC and corresponding normal lung tissues. This analysis identified 5976, 6547, and 8459 circRNAs in the NSCLC samples, compared with 6254, 4821, and 5419 circRNAs in the corresponding normal samples. Following the criteria of the circRNAs with log2(fold-change) > 1.0 and *p*-value < 0.05 were considered to be up-regulated, and those with log2(fold-change) < − 1.0 and *p*-value < 0.05 were considered to be down-regulated. 324 circRNAs were found to be differentially expressed: 228 were upregulated and 96 were downregulated (Fig. 1A). The six most significantly upregulated circRNAs were further examined based on their fold-change (Fig. 1B). To mitigate potential “Type I” errors in RNA-seq data, a heatmap depicted the expression variations of these circRNAs across four NSCLC cell lines compared with the normal alveolar epithelial cell line BEAS-2B (Fig. 1C). Notably, circ_0007386 (chr2:36668400–36,669,878) showed significant upregulation in the analyzed data. This upregulation was also observed in NSCLC cell lines (A549, H1299, H838, H1975) in comparison with BEAS-2B (Fig. 1D). A549 and H1299 cells, displaying higher expression levels of circ_0007386, were selected for subsequent investigations. RT-qPCR analysis of 60 NSCLC tissue pairs revealed higher expression of circ_0007386 in tumors compared with adjacent normal tissues, aligning with RNA-seq data results (Fig. 1E–F). The circBase database indicates that circ_0007386, located on chromosome 2 and derived from exons 3–4 back splicing of the *CRIM1* gene, forms a closed loop structure. Sanger sequencing verified the expected head-to-tail splicing of circ_0007386, confirming its predicted size and splicing site (Fig. 1G). Compared with its linear counterpart, *CRIM1* mRNA, circ_0007386 demonstrated enhanced stability, as evidenced by treatment with actinomycin D and RNase R (Fig. 1H–I). Nuclear-cytoplasmic fractionation and fluorescence in situ hybridization (FISH) revealed that circ_0007386 predominantly resides in the cytoplasm of NSCLC cells (Fig. 1J-K). These findings suggest that circ_0007386 is an upregulated and highly stable circRNA present in the cytoplasm of NSCLC cells.

### Circ_ 0007386 promotes cell proliferation in non-small cell lung cancer cells in vitro and in vivo

**Fig. 2:**
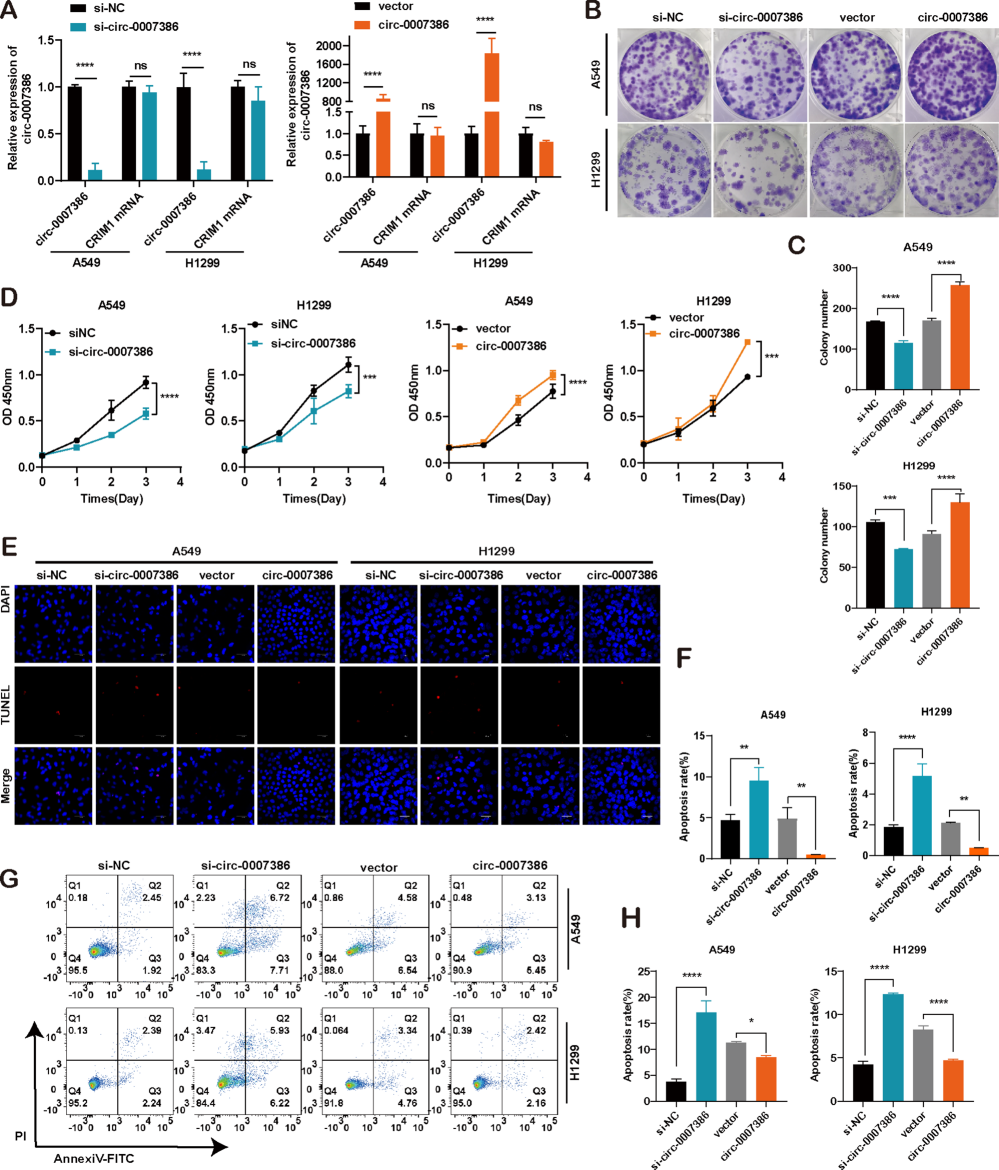
Circ_0007386 promotes the proliferation and inhibits apoptosis of NSCLC cells in vitro. **A.** The expression levels of circ_0007386 and *CRIM1* mRNA were assessed in A549 and H1299 cells after transfection with circ_0007386 siRNA and overexpression vector (si-circ_0007386 and circ_0007386) alongside the controls (si-NC and vector). **B-D.** CCK8 and colony formation assays were performed to determine cell proliferation in the indicated cell lines. **E-H.** TUNEL staining assay and flow cytometry were used to evaluate the effect on apoptosis in A549 and H1299 cells after circ_0007386 knockdown or overexpression treatment. Images are shown at 400× magnification. Scale bar = 40 μm. Data are shown as the means ± SD. *p < 0.05, **p < 0.01, ***p < 0.001, ****p < 0.0001

To evaluate the biological function of circ_0007386, both in vitro functional assays and in vivo animal experiments were conducted. siRNA and overexpression plasmids for circ_0007386 targeting its reverse splicing site were constructed and transfected into A549 and H1299 NSCLC cells. The effectiveness of circ_0007386 knockdown and upregulation was verified in these cells, showing no effect on the expression of the host gene *CRIM1* (Fig. 2A). Cell proliferation was assessed through CCK8 and cloning formation assays. Results indicated that reduction of circ_0007386 levels led to decreased cell proliferation, whereas its overexpression significantly enhanced cell growth (Fig. 2B–D, Fig. S2A). Additionally, TUNEL assays and flow cytometry revealed that silencing circ_0007386 promoted cell apoptosis, and its upregulation produced the opposite effect (Fig. 2E-H). These in vitro findings strongly suggest a carcinogenic role of circ_0007386 in NSCLC cells.

**Fig. 3:**
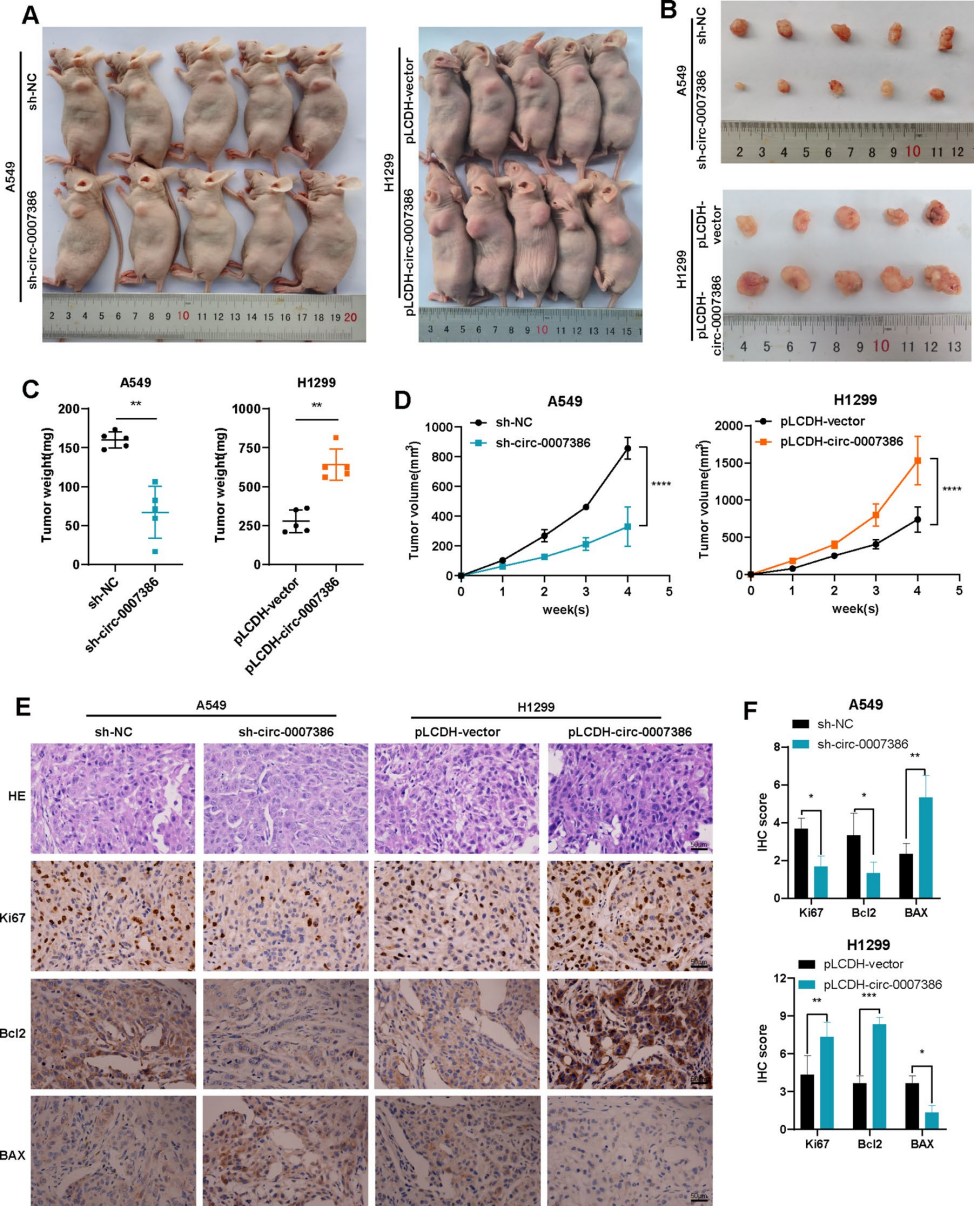
Circ_0007386 affects tumorigenesis of NSCLC cells in vivo. **(A)** Representative images of subcutaneous tumor nude mouse model, showing hypodermic injection of cells stably transfected with lentivirus expressing circ_0007386 knockdown (sh-circ_007386) or overexpression construct (pLCDH-circ_0007386) and their controls (sh-NC and pLCDH-vector) to establish subcutaneous xenograft tumors. **(B)** Images of subcutaneous xenograft tumors (*n* = 5 per group). **(C)** Tumor weight was assessed for each group. **(D)** Tumor volume was measured every week. **E-F.** HE, Ki67, Bcl2, and BAX IHC staining of xenograft tumors, with images shown at 400× magnification. Scale bar = 50 μm. Data are shown as the means ± SD. *p < 0.05, **p < 0.01, ***p < 0.001, ****p < 0.0001

To further investigate the impact of circ_0007386 on cell proliferation in vivo, a subcutaneous nude mouse tumor model was created using A549 and H1299 stably transfected with pLCDH circ_0007386 or pLCDH vector, along with sh-circ_0007386 or sh-NC. Tumors overexpressing circ_0007386 exhibited increased growth, weight, and volume compared with the vector control group, while silencing circ_0007386 yielded opposite outcomes (Fig. 3A–D, Fig. S1A–D). IHC staining of tumor tissues showed increased Ki67 and Bcl2 expression and decreased BAX expression in the overexpression group, while the opposite trends were observed in the knockdown group. (Fig. 3E-F, Fig. S1E-F). These findings further corroborate that circ_0007386 promotes the proliferation of NSCLC cells both in vitro and in vivo.

### Circ_0007386 functions as a sponge for miR-383-5p

CircRNAs are a class of non-coding RNAs crucial for regulating gene expression. A key regulatory mechanism of circRNAs involves acting as miRNA sponges, competing with endogenous RNAs for miRNA binding [20].

**Fig. 4:**
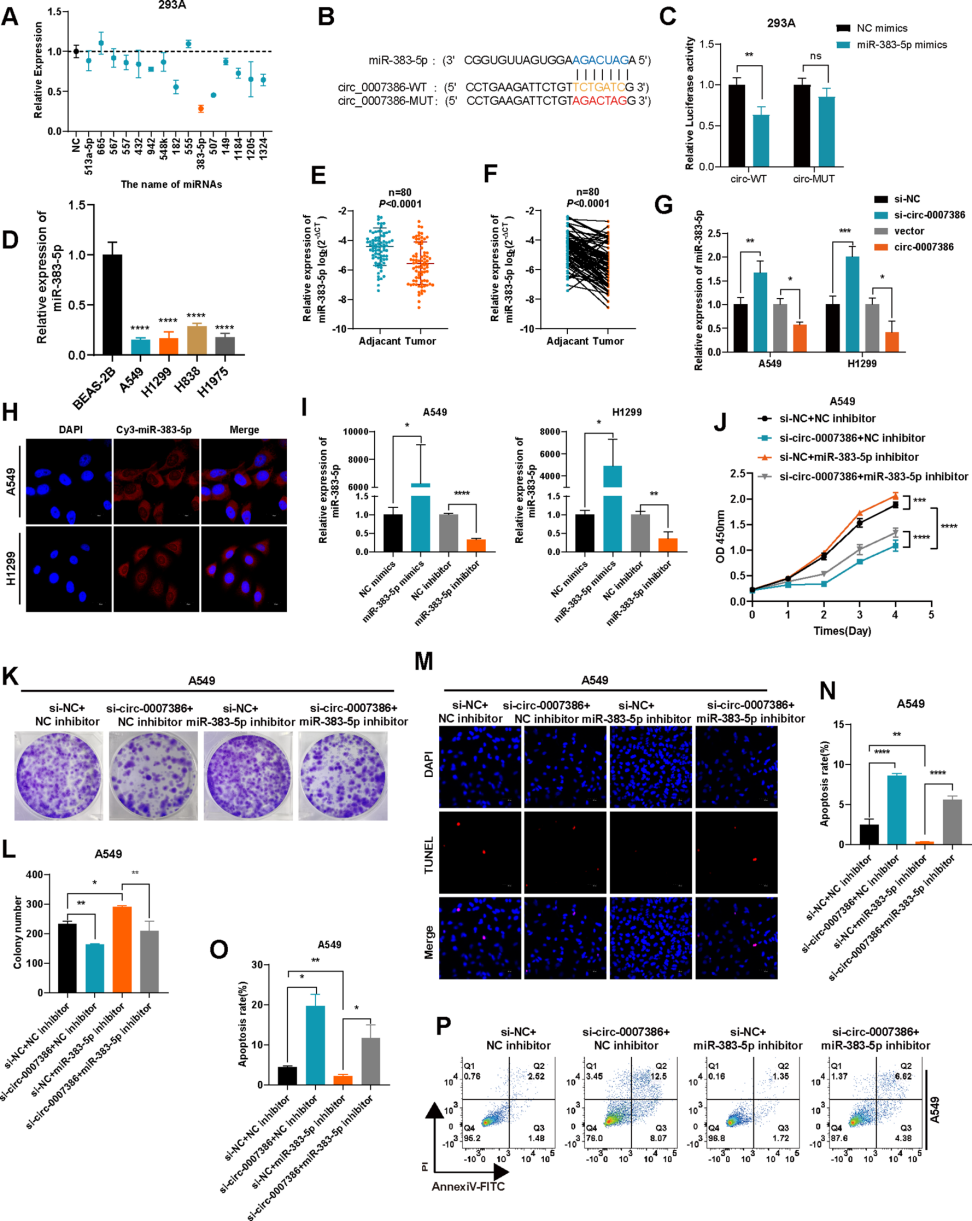
Circ_0007386 acts as a sponge for miR-383-5p. **(A)** Relative luciferase activity of the circ_0007386 wild-type (circ_0007386 WT) in 293 A cells transfected with 15 candidate miRNAs mimics or mimic NC. **(B)** Schematic of the luciferase reporter plasmids of circ_0007386 WT and mutant-type (circ_0007386 MUT). **(C)** Luciferase activity of circ_0007386 WT and circ_0007386 MUT transfected with miR-383-5p mimic or mimic NC. **(D)** Relative expression levels of miR-383-5p were determined using RT-qPCR in various cell lines of NSCLC. **E-F.** Relative expression of miR-383-5p in NSCLC tissues and adjacent nontumor tissues was analyzed using RT-qPCR (*n* = 80). **G.** Relative expression of miR-383-5p in A549 and H1299 cells, transfected as indicated, was detected by RT-qPCR. **H.** FISH assay showed that miR-383-5p (Cy3-labeled probe) was abundant in the cytoplasm, and DAPI was used to stain the nucleus. Images are shown at 600× magnification. Scale bar = 20 μm. **I.** RT-qPCR of the relative expression levels of miR-383-5p in A549 and H1299 cells transfected with the miR-383-5p mimics or inhibitor or its controls. **J-L.** Rescue experiments, including CCK8 and colony formation were conducted in cells treated with four different conditions (si-NC + inhibitor NC, si-circ_0007386 + inhibitor NC, si-NC + miR-383-5p inhibitor, si-circ_0007386 + miR-383-5p inhibitor). **M-P.** Rescue experiments, including TUNEL staining and flow cytometry assays were conducted in A549 and H1299 cells in the indicated groups. Images are shown at 400× magnification. Scale bar = 40 μm. Data are shown as the means ± SD. *p < 0.05, **p < 0.01, ***p < 0.001, ****p < 0.0001

This study aimed to explore if circ_0007386 operates as a miRNA sponge by targeting specific miRNAs. Using the Circinteractome prediction database [21], we identified 15 candidate miRNA targets for circ_0007386 (Table S4). Dual luciferase reporter assays were conducted by co-transfecting cells with a luciferase reporter plasmid containing the circ_0007386 (circ_0007386 WT) sequence and each of the 15 candidate miRNAs in 293 A cells. Notably, the co-transfection with miR-383-5p resulted in the most pronounced reduction in luciferase activity, when miR-383-5p was co-transfected with circ_0007386 WT (Fig. 4A) leading us to further investigate miRNA-383-5p. Additionally, we constructed a mutant luciferase reporter plasmid (circ_0007386 MUT) with alterations in the presumed miR-383-5p binding site (Fig. 4B). The co-transfection of circ_0007386 MUT with miR-383-5p did not result in significant luciferase activity changes, underscoring the specificity of their interaction (Fig. 4C). We observed a notably reduced expression of miR-383-5p in NSCLC cell lines and tissues (Fig. 4D–F), and manipulating circ_0007386 levels inversely affected miR-383-5p expression, supporting a competitive endogenous RNA mechanism (Fig. 4G). FISH analysis demonstrated the cytoplasmic presence of miR-383-5p in A549 and H1299 cells, similar to that of circ_0007386 (Fig. 4H). Co-localization analysis were performed to validate the interactions between circ_0007386 and miR-383-5p. The results indicated that circ_0007386 and miR-383-5p were localized primarily in the cytoplasm (Fig. S2B). Co-localization analysis performed using Image J software confirmed the spatial co-expression of circ_0007386 with miR-383-5p (Fig. S2C) (Pearson correlation coefficient *r* = 0.71). Examining miR-383-5p’s role in NSCLC, we modulated its expression using mimics and inhibitors in A549 and H1299 cells, as confirmed by RT-qPCR (Fig. 4I). The proliferation of these cells was enhanced by miR-383-5p inhibitors and mitigated the suppressive effect of si-circ_0007386, as determined by CCK8, colony formation, TUNEL assay, and flow cytometry assays (Fig. 4J–P, S2D–H). Co-transfection experiments with miR-383-5p mimics and circ_0007386 plasmids in A549 and H1299 cells produced analogous outcomes (Fig. S2I–M), suggesting that miR-383-5p could function as a downstream tumor suppressor mediated by circ_0007386.

### CIRBP is a target of mir-383-5p and indirectly regulated by circ_0007386

**Fig. 5:**
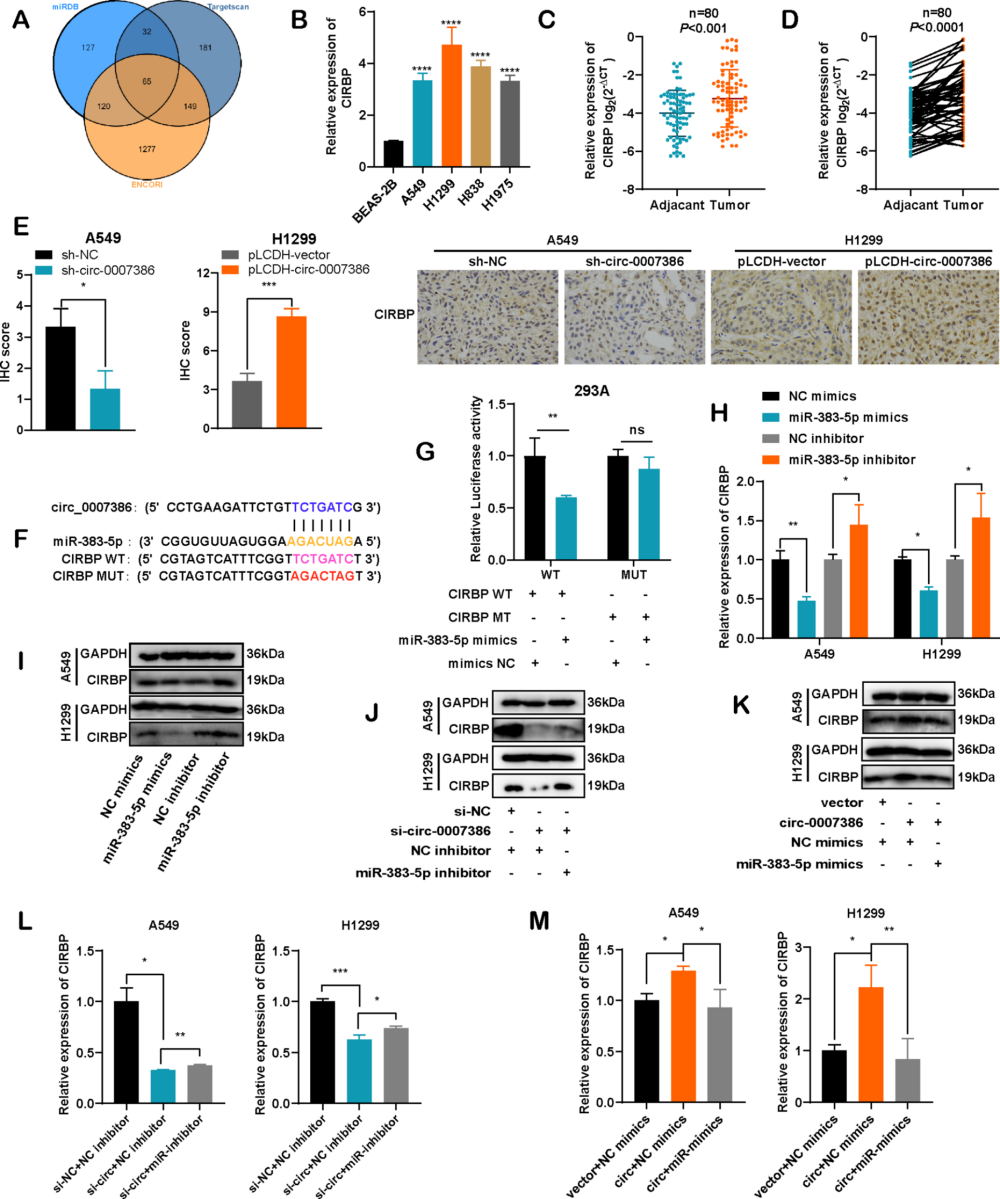
CIRBP is a target gene of miR-383-5p and indirectly regulated by circ_0007386. **A**. Venn diagram showing 65 genes that are potential targets of miR-383-5p predicted by three databases (Targetscan, miRDB, and ENCORI). **B.** The expression of CIRBP mRNA in NSCLC cells was assessed using RT-qPCR. **C-D.** The relative expression levels of CIRBP were compared between NSCLC tissues and adjacent nontumor tissues (*n* = 80). **E.** IHC staining of CIRBP expression in xenograft tumors in four different groups (circ_0007386 overexpression construct (pLCDH-circ_0007386), control vector (pLCDH-vector), circ_0007386 shRNA (sh-circ_0007386) and control shRNA (sh-NC)). Images are shown at 400× magnification. Scale bar = 50 μm. **F.** Schematic of the luciferase reporter plasmids for CIRBP wild-type (CIRBP WT) and mutant-type (CIRBP MUT). **G.** Luciferase activity of the CIRBP WT and CIRBP MUT reporters in 293 A cells transfected with miR-383-5p mimics or mimic NC. **H-I.** RT-qPCR and western blot of CIRBP mRNA and protein levels in cells transfected with miR-383-5p mimics and inhibitor and its controls. **J-M.** Relative expression of CIRBP at both the mRNA and protein levels in the indicated groups (si-NC + NC-inhibitor, si-circ_0007386 + NC-inhibitor, si-circ_0007386 + miR-383-5p inhibitor and vector + mimics NC, circ_0007386 + NC-mimics, circ_0007386 + miR-383-5p mimics). Data are shown as means ± SD. *p < 0.05, **p < 0.01, ***p < 0.001, ****p < 0.0001

To elucidate the mechanism behind circ_0007386’s influence on the miR-383-5p regulatory axis, we conducted an extensive cross-analysis using three gene prediction databases: TargetScan, miRDB, and ENCORI. This investigation revealed 65 potential overlapping genes that may be targeted by miR-383-5p (Fig. 5A). Upon reviewing the literature on these candidates, we selected CIRBP for further research. Previous studies have shown that CIRBP is regulated by miR-383-5p [22] and facilitates the proliferation of cancer cells [5, 23]. This finding aligns with the observed expression patterns of circ_0007386 in NSCLC cell lines and tissues, where CIRBP levels were higher in tumor samples compared with normal counterparts (Fig. 5B–D). IHC staining of CIRBP in subcutaneous tumors from mice further confirmed the correlation between circ_0007386 and CIRBP expression (Fig. 5E). Analysis based on the data from the databases mentioned earlier revealed sequence complementarity between the CIRBP 3’UTR and miR-383-5p. To verify this, we engineered luciferase reporter plasmids for both wild-type (CIRBP WT) and mutant (CIRBP MUT) CIRBP 3’UTR, demonstrating that CIRBP is indeed directly targeted by miR-383-5p (Fig. 5F). Co-transfection with miR-383-5p mimics in 293 A cells significantly reduced luciferase activity from the CIRBP WT reporter but not from the CIRBP MUT reporter (Fig. 5G). Moreover, RT-qPCR and Western blot analyses demonstrated that miR-383-5p markedly reduced CIRBP expression levels, while inhibitors of miR-383-5p significantly increased them (Fig. 5H–I, Fig. S5A). Furthermore, circ_0007386 was found to positively influence the mRNA and protein levels of CIRBP, effects that were partially reversed by the use of miR-383-5p mimics or inhibitors (Fig. 5J–M, Fig. S5B-C). These results collectively suggest that CIRBP is a downstream target of miR-383-5p, with its regulation being indirectly regulated by circ_0007386.

Circ_0007386 affects the proliferation ability of NSCLC cells through miR-383-5p/CIRBP axis.

**Fig. 6:**
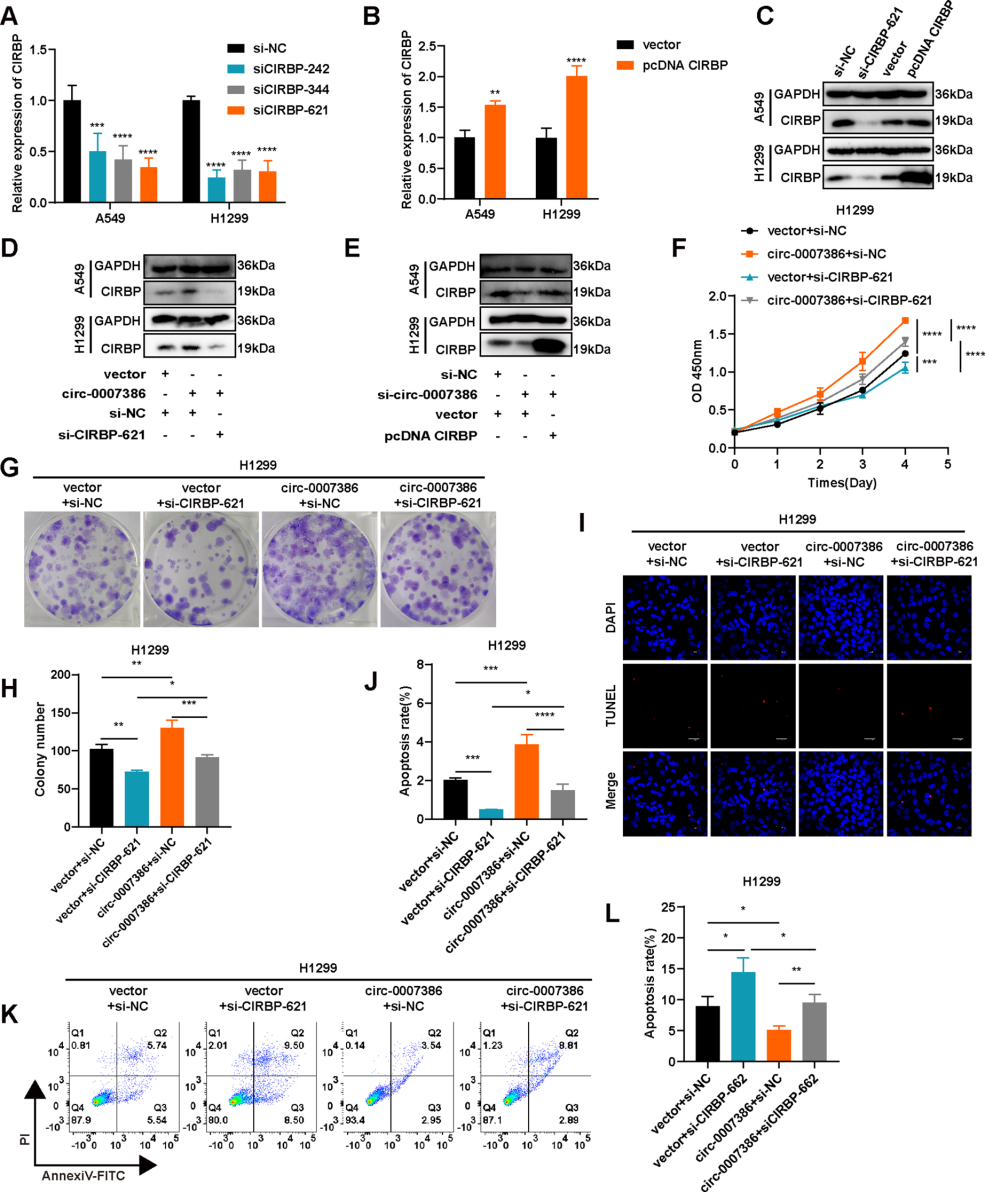
Circ_0007386 regulates proliferation and apoptosis in NSCLC cells via the miR-383-5p/CIRBP axis. **A-C.** The relative expression of CIRBP mRNA and protein was assessed in cells transfected with three short hairpin RNAs (si-CIRBP) and an overexpression vector of CIRBP (pcDNA CIRBP). **D-E.** Western blot analysis of CIRBP in cells across indicated groups (vector + si-NC, circ_0007386 + si-NC, circ_0007386 + si-CIRBP-621 and si-NC + vector, si-circ_0007386 + vector, si-circ_0007386 + pcDNA CIRBP). **F-H.** Rescue experiments, including CCK8 and colony formation were performed in the four treatment groups shown (vector + si-NC, vector + si-CIRBP-621, circ_0007386 + si-NC, circ_0007386 + si-CIRBP-621). **I-L.** Rescue experiments, including TUNEL staining and flow cytometry assays were conducted in A549 and H1299 cells in the indicated groups. Images are shown at 400× magnification. Scale bar = 40 μm. Data are shown as means ± SD. *p < 0.05, **p < 0.01, ***p < 0.001, ****p < 0.0001

To investigate the roles of CIRBP in NSCLC cells, we altered CIRBP levels with three siRNAs and an overexpression plasmid, validating the modifications in mRNA and protein levels in A549 and H1299 cells (Fig. 6A–C, Fig. S6A). Our findings also showed that circ_0007386 can influence CIRBP protein expression (Fig. 6D–E, Fig. S6B-C). Subsequently, we conducted functional assays to evaluate the impact on cellular behavior. Knockdown of CIRBP in A549 and H1299 cells resulted in decreased cell proliferation and increased apoptosis (Fig. 6F–L, S3A–E), whereas CIRBP overexpression inhibited apoptosis (Fig. S3F-J). Additionally, the enhancement in proliferation observed with circ_0007386 overexpression in NSCLC cells was diminished by CIRBP knockout (Fig. 6F–J). Conversely, silencing circ_0007386 led to a decrease in the upregulated CIRBP expression associated with enhanced proliferation (Fig. S3F-J). These findings suggest that circ_0007386 promotes carcinogenesis in NSCLC through the miR-383-5p/CIRBP axis.

### Circ_0007386 promotes proliferation via the miR-3835p/CIRBP axis through a PI3K/AKT signaling pathway

**Fig. 7:**
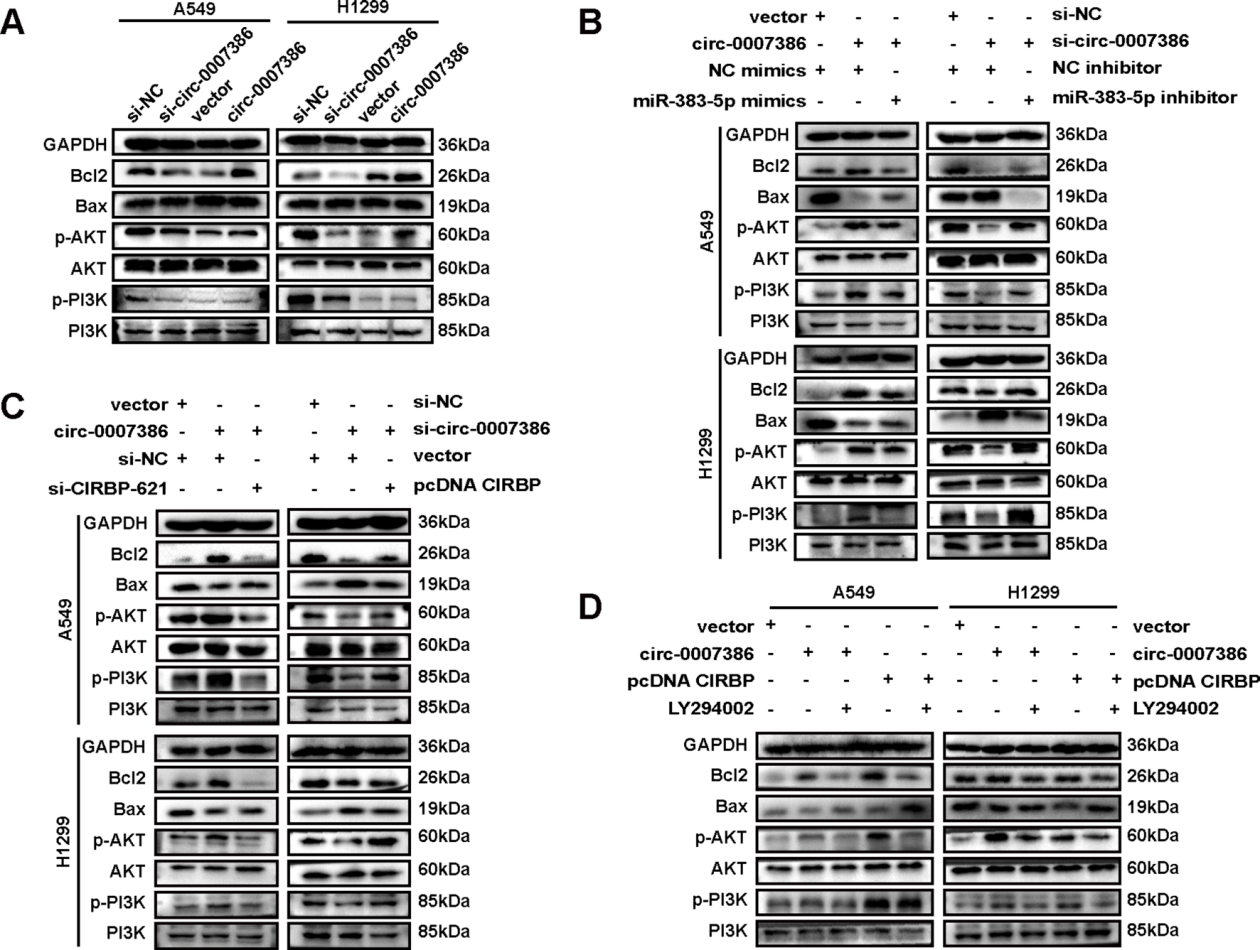
Circ_0007386 via the miR-383-5p/CIRBP axis promotes proliferation through a PI3K/AKT signaling pathway. **A.** Western blot analysis of p-PI3K, PI3K, p-AKT, AKT, and apoptosis-associated molecules in cells with either circ_0007386 knockdown or overexpression. **B-C.** Western blot analysis of p-PI3K, PI3K, p-AKT, AKT, and apoptosis-associated molecules in cells treated as indicated. **D.** Western blot analysis of p-PI3K, PI3K, p-AKT, AKT, and apoptosis-associated molecules in cells transfected with the indicated vectors and treated with the PI3K/AKT pathway inhibitor (LY294002)

To investigate the role of the circ_0007386/miR-383-5p/CIRBP regulatory axis in the proliferation and apoptosis of NSCLC cells, we assessed the expression of apoptosis markers through western blot analysis. Silencing circ_0007386 resulted in elevated BAX levels and reduced expression of Bcl2, p-PI3K, and p-AKT, whereas circ_0007386 overexpression exhibited an inverse pattern (Fig. 7A, Fig. S7A). These effects were negated by employing miR-383-5p inhibitors and mimics (Fig. 7B, Fig. S7B), alongside adjustments in CIRBP expression (Fig. 7C, Fig. S7C).

To further explore the role of the PI3K/AKT pathway, we applied LY294002, a potent and highly selective inhibitor [24]. Notably, overexpression of circ_0007386 and CIRBP resulted in a decrease in BAX protein levels and an increase in the expression of Bcl2, p-PI3K, and p-AKT, which were subsequently reversed by LY294002 treatment (Fig. 7D, Fig. S7D). In conclusion, our data reinforce the evidence that the circ_0007386/miR-383-5p/CIRBP axis significantly impacts apoptosis in NSCLC cells via the PI3K/AKT signaling pathway.

### YAP1 regulates alternative splicing of *CRIM1* pre-mRNA under hypoxic conditions

**Fig. 8:**
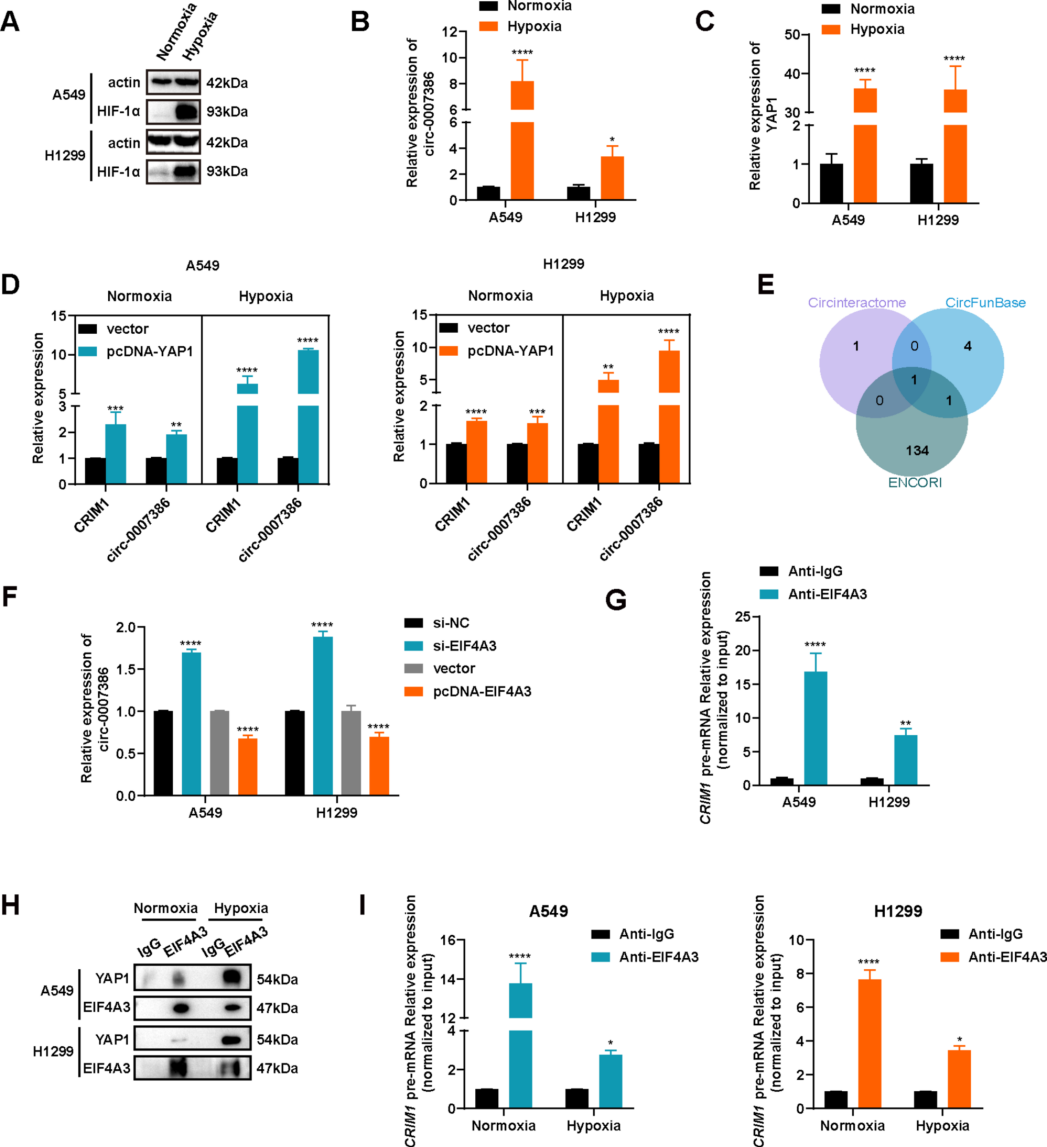
Hypoxia-enhanced YAP1-EIF4A3 interaction drives circ_0007386 circularization by competing with *CRIM1* pre-mRNA linear splicing. **(A)** Western blot analysis of HIF1α expression in cells under hypoxia or normoxia. **(B)** circ_0007386 expression under hypoxia and normoxia analyzed using RT-qPCR. **(C)** YAP1 expression under hypoxia and normoxia analyzed by RT-qPCR. **(D)** The relative expression of *CRIM1* mRNA and circ_0007386 after YAP1 overexpression under hypoxia and normoxia were detected by RT-qPCR. **(E)** Venn diagram showing RBPs that bind to the *CRIM1* pre-mRNA transcript predicted by three databases (CircInteractome, CircFunBase, and ENCORI). **(F)** Relative expression of circ_0007386 in A549 and H1299 cells transfected with the indicated vectors or siRNAs was determined using RT-qPCR. **(G)** RIP assays using an EIF4A3 antibody were performed to examine interactions with *CRIM1* mRNA. **(H)** CoIP analysis of the YAP1-EIF4A3 interaction in cells treated as indicated. **(I)** RIP assays using an EIF4A3 antibody were performed to examine interactions with *CRIM1* mRNA under hypoxia and normoxia. Data are shown as the means ± SD. *p < 0.05, **p < 0.01, ***p < 0.001, ****p < 0.0001

Hypoxia frequently occurs alongside tumor progression [13]. To explore the role of hypoxia in this interaction, we conducted experiments on NSCLC cells under hypoxic conditions. Initially, we established a hypoxia model in cells, evidenced by increased HIF1α expression under hypoxic compared with normoxic conditions (Fig. 8A). Despite observing high levels of circ_0007386 in NSCLC cells cultured under normoxia (Fig. 1D), we observed a significant rise in circ_0007386 levels following hypoxia treatment (Fig. 8B). This suggests that hypoxia facilitates the formation of circ_0007386. Consequently, we investigated the mechanism behind the enhanced formation of circ_0007386 under hypoxia. Previous studies have indicated that YAP1 can enhance the expression of the circ_0007386 host gene *CRIM1* [25, 26]. RT-qPCR analysis revealed increased YAP1 expression in cells treated with hypoxia (Fig. 8C). Modifying YAP1 expression in cells (Fig. S4A–B) indicated that YAP1 overexpression equally raised the expression of *CRIM1* and circ_0007386 (Fig. 8D). However, under hypoxia, circ_0007386 expression increased significantly more than *CRIM1* mRNA (Fig. 8D). Conversely, YAP1 knockdown resulted in a reverse expression pattern of *CRIM1* and circ_0007386 (Fig. 8E, S4C–D). These findings suggest that YAP1 may preferentially induce exon circularization of *CRIM1* pre-mRNA to form circ_0007386 over canonical linear splicing under hypoxic conditions.

### Hypoxia-enhanced YAP1-EIF4A3 interaction relieves the inhibition of circ_0007386 biogenesis by interrupted EIF4A3-binding *CRIM1* pre-mRNA

Prior studies have shown that RBPs affect the biogenesis of circRNAs by binding with the intronic regions adjacent to the exons that circularize, thereby augmenting or hindering the reverse splicing process [14, 27–29]. To explore whether RBPs regulated the formation of circ_0007386, we utilized three online prediction tools: CircInteractome, CircFunBase, and ENCORI. These analyses revealed that EIF4A3, with multiple binding sites in the introns flanking the circularized exons of *CRIM1* pre-mRNA, could be the crucial RBP influencing circ_0007386 formation (Fig. 8F, S4H). To confirm the regulatory relationship of EIF4A3 and circ_0007386, we analyzed circ_0007386 expression following EIF4A3 modulation using siRNAs and an overexpression plasmid (Fig. S4E–F). The results demonstrated that EIF4A3 knockdown increased circ_0007386 expression, whereas EIF4A3 overexpression had the reverse effect (Fig. 8G). Subsequent RIP analysis indicated that EIF4A3 binds to *CRIM1* pre-mRNA (Fig. 8H, S4G), suggesting that EIF4A3 impedes circ_0007386 formation potentially by binding to *CRIM1* pre-mRNA. Moreover, CoIP experiments confirmed that YAP1 interacts with EIF4A3, and this interaction is intensified under hypoxic conditions (Fig. 8I). Additional RIP analysis demonstrated that hypoxia reduces EIF4A3’s binding to *CRIM1* pre-mRNA (Fig. 8J). In summary, our results suggest that higher levels of YAP1 expression under hypoxic conditions facilitates circ_0007386 by competing with *CRIM1* pre-mRNA for EIF4A3 binding.

### Circ_0007386/miR-383-5p/CIRBP axis correlates with NSCLC prognosis

**Fig. 9:**
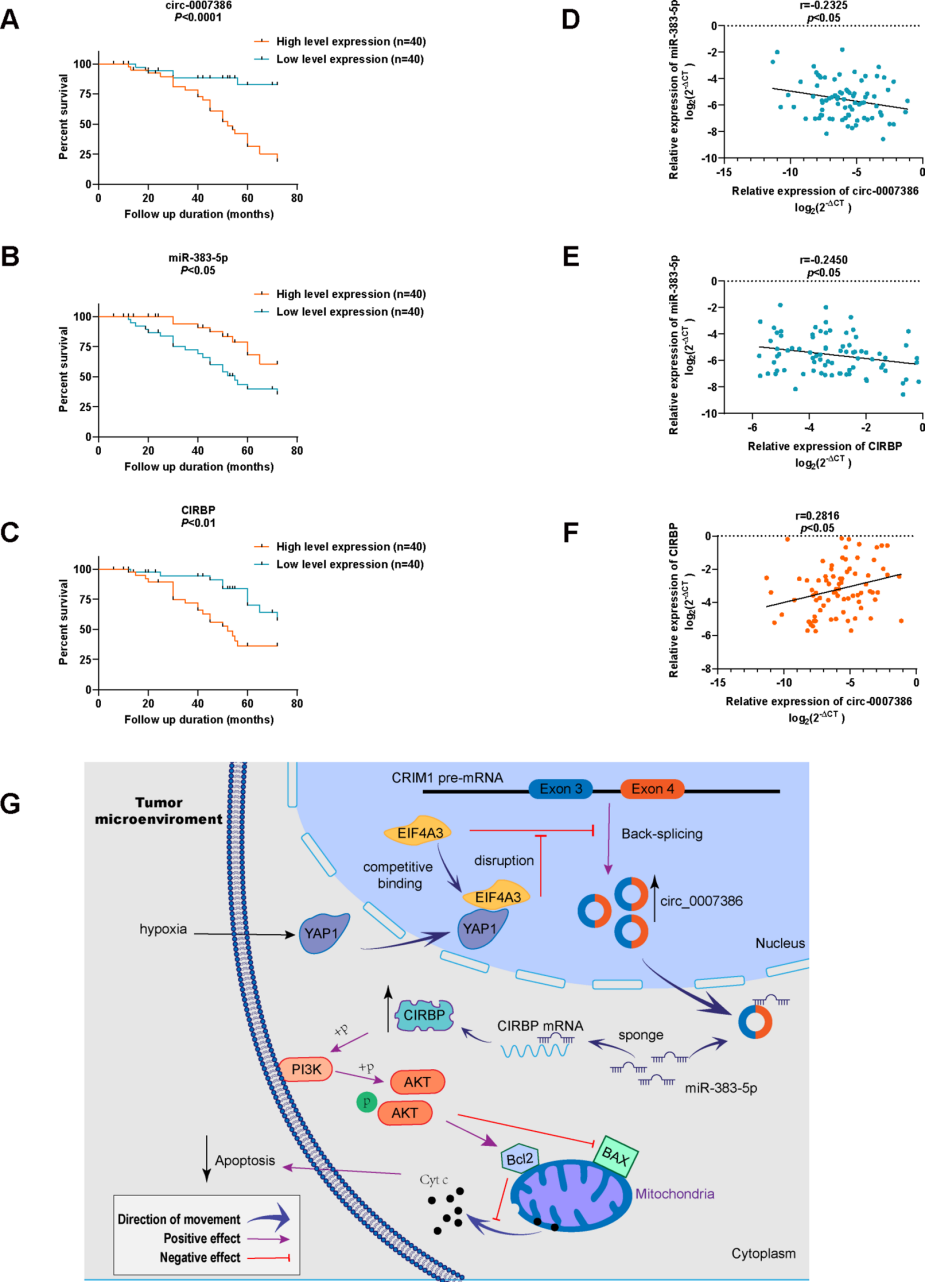
Circ_0007386/miR-383-5p/CIRBP axis correlates with NSCLC prognosis and the schematic diagram illustrates the mechanism. **A**-**C**. Kaplan–Meier survival plots for NSCLC patients based on low and high expressions of circ_0007386, miR-383-5p, or CIRBP (*n* = 80). **D**-**F**. Correlation analysis of circ_0007386, miR-383-5p, and CIRBP expression analyzed using RT-qPCR in NSCLC tissues (*n* = 80). **G**. Schematic representation of the underlying mechanism where hypoxia-enhanced YAP1-EIF4A3 interaction mediates circ_0007386 circularization and promotes non-small cell lung cancer progression via miR-383-5p/CIRBP axis. Data are shown as means ± SD. *p < 0.05, **p < 0.01

In our subsequent investigation, we assessed the clinical significance of circ_0007386, miR-383-5p, and CIRBP in patients with NSCLC. These patients were categorized into groups with high and low expressions based on their median expression level. Our Kaplan–Meier analysis indicated that NSCLC patients with higher expression of circ_0007386, lower expression of miR-383-5p, and higher expression of CIRBP experienced worse overall survival rates (Fig. 9A–C). Table 1 outlines the association between the expression of circ_0007386, miR-383-5p, CIRBP, and the clinical characteristics of NSCLC patients. Our analysis revealed that tumor diameter was positively correlated with high expression of circ_0007386 and negatively correlated with expression of miR-383-5p. Additionally, we found that T stage 1 was associated with high expression of CIRBP. Pearson correlation analysis demonstrated negative associations between the expression levels of circ_0007386 and miR-383-5p in NSCLC tissues and cells, as well as between miR-383-5p and CIRBP expression. Notably, a positive correlation was observed between circ_0007386 and CIRBP (Fig. 9D–F, Fig. S4I). These findings highlight the clinical significance of the circ_0007386/miR-383-5p/CIRBP axis in NSCLC.

**Table 1:**
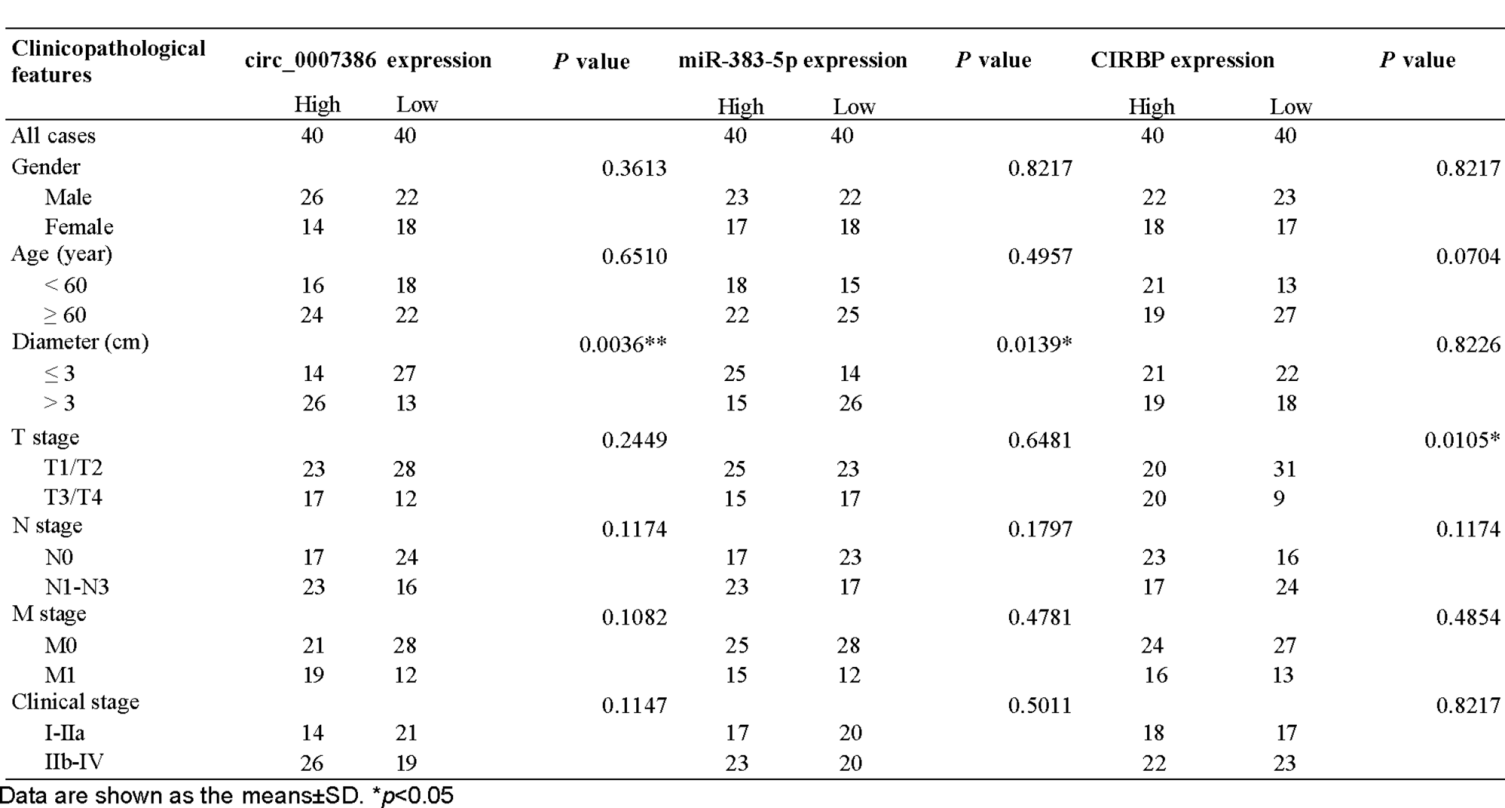
Association of circ_0007386, miR-383-5p and CIRBP expression with clinicopathological features of NSCLC (n =80)

## Discussion

Non-small cell lung cancer is a highly metastatic tumor associated with a poor prognosis, underscoring the critical need for novel biological targets to effectively identify high-risk individuals among asymptomatic patients [30, 31]. CircRNA, an endogenous RNA form prevalent in eukaryotic cells, features a covalent loop structure that confers significant resistance to RNase activity [11, 32]. Extensive research has highlighted the pivotal role of circRNA in the development and progression of various human cancers, suggesting its potential as a valuable diagnostic and prognostic biomarker [9]. For example, Wu et al. explored the role of circ_TADA2A in osteosarcoma progression and metastasis through the mir-203a-3p/CREB3 axis [33]. In another study, Luo et al. identified cir_0000190 as a contributor to tumorigenesis and immune evasion in NSCLC by upregulating PD-L1 expression [34]. Similarly, Wu et al. found that circ_0017639 increases the proliferation, migration, and invasion capabilities of NSCLC cells by activating the PI3K/AKT pathway [35]. These findings highlight the potential of circRNAs as promising targets for enhancing cancer detection, prognosis, and therapeutic interventions [36]. This study focuses on circ_0007386, identified for its elevated expression levels in NSCLC. We determined that circ_0007386 contributes to NSCLC proliferation and inhibits apoptosis. Moreover, our results validate circ_0007386’s utility as a biomarker for diagnosing and prognosticating NSCLC outcomes.

The biological role of circRNA as a miRNA sponge has received considerable attention in scientific literature [37]. Specifically, circ-0003258 has been shown to promote prostate cancer metastasis by sponging miR-653-5p and interacting with IGF2BP3 [38]. Conversely, circ-PLEKHM3 has been identified as an inhibitor of ovarian cancer progression by competitively sponging endogenous miR-320a [39]. In our study, we utilized a dual-luciferase reporter assay and FISH analysis to confirm the binding of miR-383-5p to circ_0007386. Our findings align with previous reports on miR-383-5p [40], demonstrating its role in promoting apoptosis and inhibiting proliferation in NSCLC. The rescue experiments further corroborated circ_0007386’s role as a sponge for miR-383-5p NSCLC progression.

Previous research indicated that miR-383-5p promotes apoptosis of ovarian granulosa cells by targeting CIRBP [22]. Through bioinformatics analysis, we identified CIRBP as a potential target gene. Initially, an upregulation of CIRBP was noted in both NSCLC tissues and cell lines, paralleling the expression pattern of circ_0007386. Subsequent double luciferase assays confirmed CIRBP as a direct target of miR-383-5p. RT-qPCR and Western blot analyses revealed a direct correlation between CIRBP and circ_0007386 expression and an inverse relationship with miR-383-5p levels. Co-transfection of circ_0007386 and miR-383-5p in cells mitigated the downregulation of CIRBP by miR-383-5p. In functional assays, CIRBP was shown to reverse the suppression of proliferation and to enhance apoptosis following circ_0007386 knockdown. Conversely, CIRBP overexpression had the opposite effect. These findings suggest that the circ_0007386/miR-383-5p/CIRBP axis plays a critical role in the progression of NSCLC.

The members of the PI3K play an essential role in various cellular regulatory processes, including cell proliferation, metabolism, and migration [41, 42]. Our analysis revealed that the overexpression of circ_0007386, in comparison with the control group, resulted in the increased expression of Bcl2, p-PI3K, and p-AKT, alongside a decrease in BAX expression. Similar results were observed with CIRBP overexpression, and these effects were negated by the PI3K pathway inhibitor, LY294002. Therefore, our findings strongly suggest that circ_0007386 enhances the proliferation and suppresses the apoptosis of NSCLC cells by activating the PI3K/AKT signaling pathway through the miR-383-5p/CIRBP axis.

Hypoxia is recognized for its crucial role in cancer growth and active regulation of gene expression [13, 43]. In a hypoxic microenvironment, HIF1⍺ induced the transcription of circ-CDYL, thereby promoting the lung metastasis of circulating cancer stem cells [44]. Additionally, hypoxia was found to promote chemotherapy resistance by downregulating the expression of the SKA1 in human osteosarcoma [45]. Our study found that hypoxia enhances the expression of circ_0007386. Consequently, we further investigated the underlying mechanism of hypoxia in the formation of circ_0007386. Given that exon-derived circRNA and its host mRNA are produced from the same pre-mRNA, circRNA can interfere with host mRNA splicing, whereas canonical pre-mRNA splicing may compete with the exon circularization process [46, 47]. Previous studies have shown that hypoxia can stimulate the expression of YAP1 (15), and YAP1 can enhance the expression of the circ_0007386 host gene *CRIM1* [25, 26]. Our findings revealed that YAP1 not only facilitates the canonical pre-mRNA linear splicing of *CRIM1* pre-mRNA into *CRIM1* mRNA but also promotes exon circularization to form circ_0007386. Interestingly, we also found that the exon circularization process is further stimulated under hypoxic conditions, leading to a higher production of circ_0007386 compared with *CRIM1* mRNA.

Various RBPs can bind to intronic sequences adjacent to circularized exons, influencing their circularization positively or negatively (48, 49). For instance, RNA adenosine deaminase 1 (ADAR1) inhibits circRNA expression by editing A-to-I in RNA duplexes flanking circular exons, which diminishes the complementarity and stability of these RNA pairs [50]. EIF4A3, a crucial element of the exon junction complex, affects pre-mRNA splicing and thereby circRNA production [51]. Through bioinformatic analysis, we predicted and subsequently confirmed that overexpression of EIF4A3 inhibited the production of circ_0007386, whereas depletion of EIF4A3 promoted its formation. Furthermore, we demonstrated EIF4A3’s interaction with *CRIM1* pre-mRNA, indicating that EIF4A3 impedes circ_0007386 formation by binding to *CRIM1* pre-mRNA. Our previous study revealed that HIF1α collaborates with SP1 to regulate the cyclization of circ_0001875 in a hypoxic tumor microenvironment [14]. Building on this, we further explored whether the formation of circ_0007386 is mediated by the interaction mechanism of EIF4A3. Our experiments revealed a significant interaction between EIF4A3 and YAP1, particularly under hypoxic conditions and revealed that EIF4A3’s affinity for *CRIM1* pre-mRNA decreases in hypoxia. Collectively, these findings suggest that YAP1 may outcompete *CRIM1* pre-mRNA for EIF4A3 binding under hypoxic conditions, leading to the liberation of *CRIM1* pre-mRNA and an increase in circ-0007386 formation. This discovery enhances our understanding of the mechanisms underlying hypoxia-mediated circRNA biogenesis.

## Conclusion

Collectively, circ_0007386 influences cell proliferation and apoptosis by targeting the miR-383-5p/CIRBP axis via the PI3K/AKT signaling pathway. Notably, the interaction between circ_0007386, miR-383-5p, and CIRBP is significantly correlated with the clinical outcomes and prognosis of patients with NSCLC. Furthermore, EIF4A3 acts to suppress the biogenesis of circ_0007386. Under hypoxic conditions, YAP1 competes with *CRIM1* pre-mRNA for EIF4A3 binding. This competition indirectly favors the generation of circ_0007386 over the maturation of *CRIM1* mRNA. This study provides new insights into the regulatory mechanisms of circRNA formation under tumor hypoxia.

Data are shown as the means ± SD. *p < 0.05.

### Electronic supplementary material

Below is the link to the electronic supplementary material.

Supplementary Material 1

Supplementary Material 2

Supplementary Material 3

Supplementary Material 4

Supplementary Material 5

## Data Availability

All data generated or analyzed during this study are included in this published article.

## Abbreviations

circRNA: Circular RNA
miRNA: MicroRNA
NSCLC: Non-small cell lung cancer
CIRBP: Cold-inducible RNA binding protein
3′UTR: 3′-untranslated regions
HIF1α: Hypoxia-inducible factor-1α
YAP1: Yes1 associated transcriptional regulator
EIF4A3: Eukaryotic translation initiation factor 4A3
RBP: RNA-binding protein
FBS: Fetal calf serum
siRNA: Short interfering RNA
SPF: Specific pathogen-free
HE: Hematoxylin and eosin
IHC: Immunohistochemistry
DAPI: 4,6-diamidino-2-phenylindole
CCK-8: Cell count kit-8
RIP: RNA immunoprecipitation
CoIP: Co-immunoprecipitation
FISH: Fluorescent in situ hybridization
qRT-PCR: Quantitative reverse transcription polymerase chain reaction
CRIM1: Cysteine rich transmembrane BMP regulator 1
DMEM: Dulbecco’s modified Eagle’s medium
