# Supplementary material for: Hypoxia-enhanced YAP1-EIF4A3 interaction drives circ_0007386 circularization by competing with CRIM1 pre-mRNA linear splicing and promotes non-small cell lung cancer progression
Source: J Exp Clin Cancer Res. 2024 Jul 20;43:200. doi: 10.1186/s13046-024-03116-6 (PMC11264895; doi:10.1186/s13046-024-03116-6)
Supplement: Supplementary file 1 — Supplementary Material 1 [file 13046_2024_3116_MOESM1_ESM.docx]

**Supplementary material**


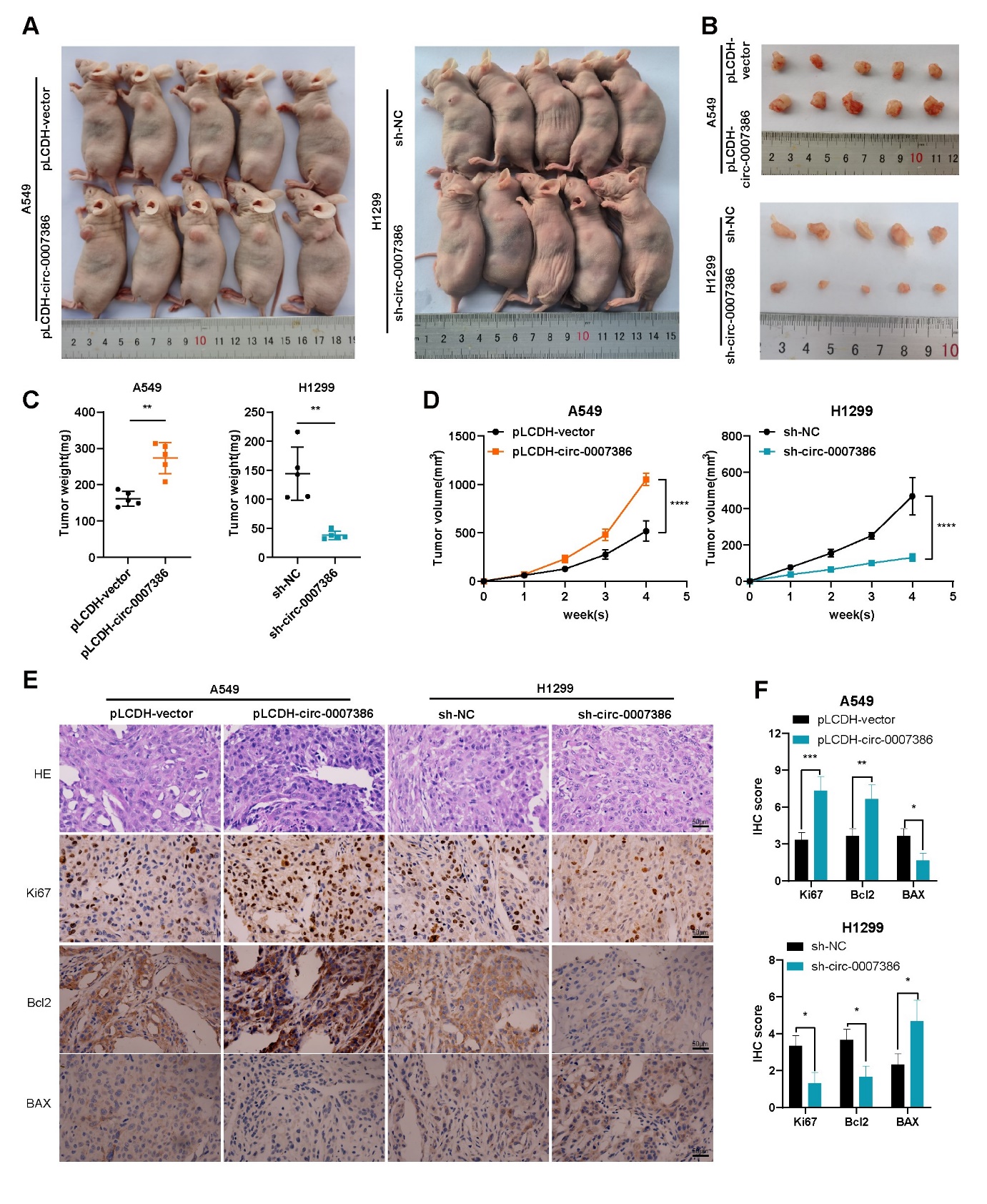


**Fig. S1 Circ_0007386 affects tumorigenesis of NSCLC cells *in vivo*. A.** Representative images of subcutaneous tumor nude mouse model. Hypodermic injection of A549 cells stably transfected with pLCDH-circ_0007386 or pLCDH vector and H1299 cells stably transfected with sh-circ_007386 or sh-NC. **B.** Images of subcutaneous xenograft tumors (n = 5 per group). **C.** Tumor weight in each group. **D.** Tumor volume was measured every week. **E-F.** HE, Ki67, Bcl2, and BAX IHC staining of xenograft tumors, with images shown at 400× magnification. Scale bar = 50 μm. Data are shown as means ± SD. **p* < 0.05, ***p* < 0.01, ****p* < 0.001, *****p* < 0.0001.


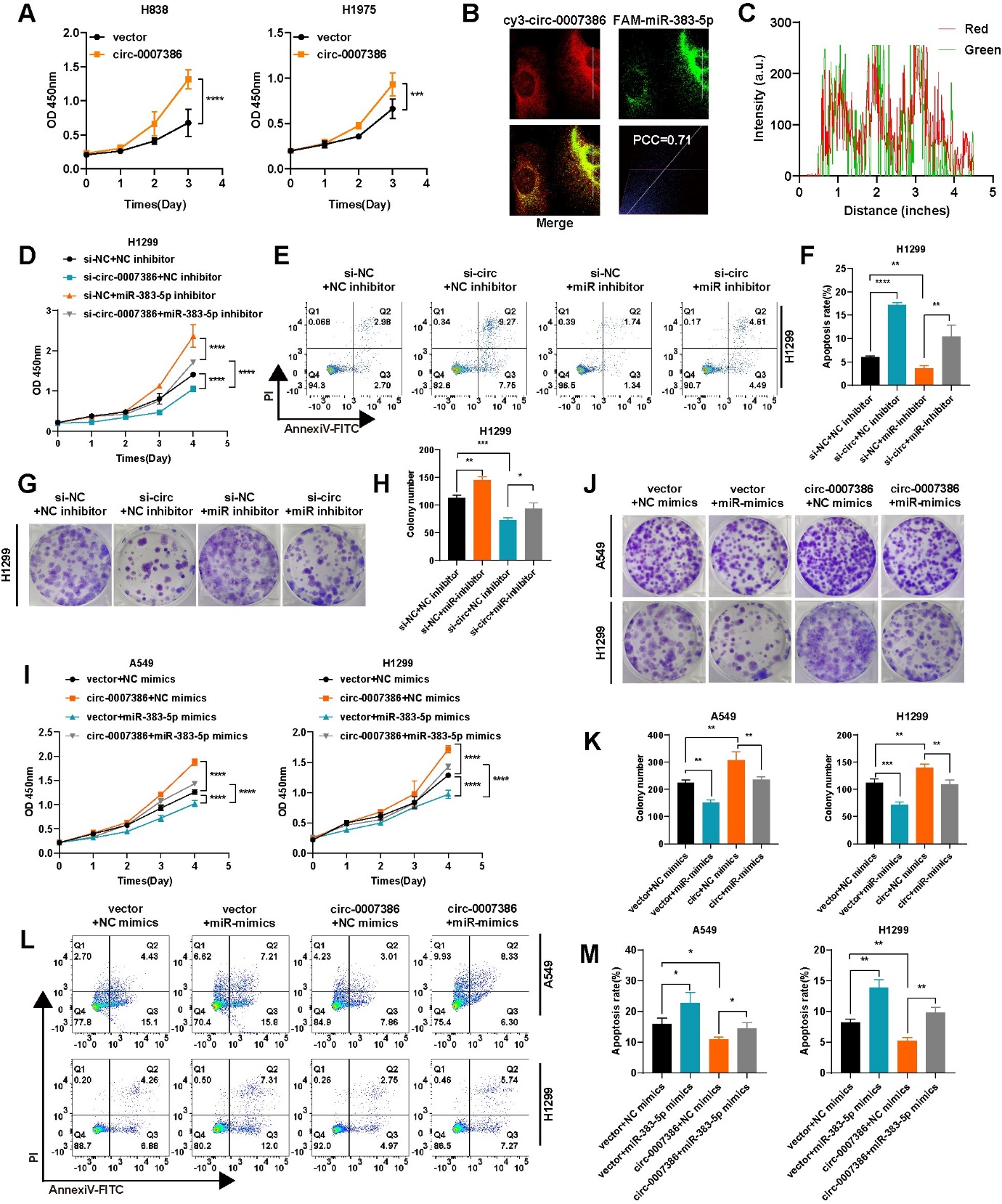


**Fig. S2 Circ_0007386 acts as a sponge for miR-383-5p. A.** CCK8 was performed to determine cell proliferation in H838 and H1975 cells. **B-C.** Co-localization of circ_0007386 (red) and miR-383-5p (green). Images are shown at 600× magnification. Scale bar = 20 μm. ImageJ was utilized to perform Pearson's correlation coefficient analysis to determine the co-localization of green and red. **D-H.** Rescue experiments. CCK8, colony formation, and flow cytometry assays were conducted in cells in four treatment groups (si-NC + inhibitor NC, si-circ_0007386 + inhibitor NC, si-NC + miR-383-5p inhibitor, si-circ_0007386 + miR-383-5p inhibitor). **I-M.** Rescue experiments. CCK8, colony formation and flow cytometry assays were conducted in cells in four treatment groups (vector + NC-mimics, vector + miR-383-5p mimics, circ_0007386 + NC-mimics, circ_0007386 + miR-383-5p mimics). Data are shown as means ± SD. **p* < 0.05, ***p* < 0.01, ****p* < 0.001, *****p* < 0.0001.


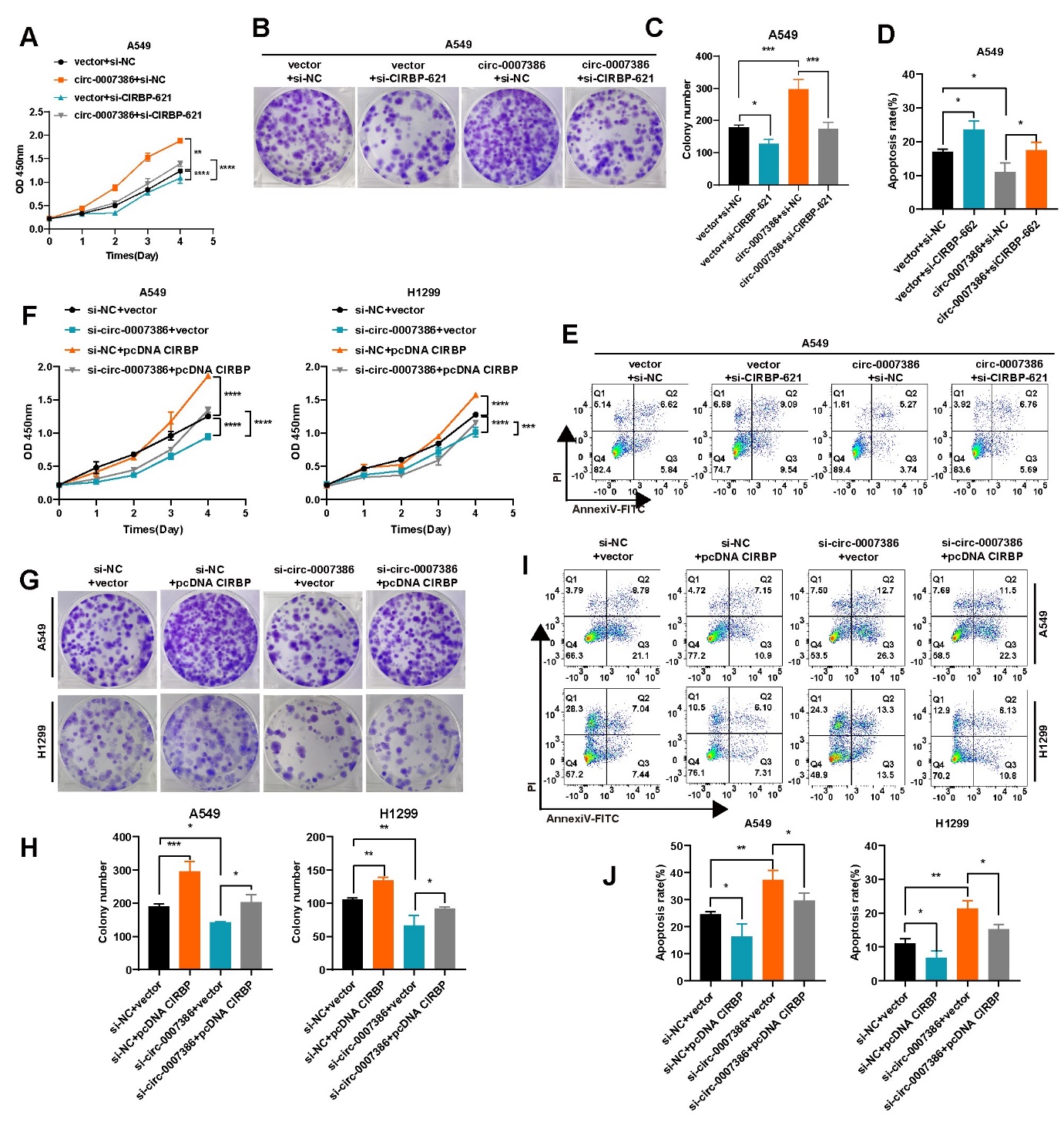


**Fig. S3 Circ_0007386 regulates proliferation and apoptosis in NSCLC cells via the miR-383-5p/CIRBP axis. A-E.** CCK8, colony formation and Flow cytometry assay in the four experimental groups shown (vector + si-NC, vector + si-CIRBP-621, circ_0007386 + si-NC, circ_0007386 + si-CIRBP-621). **F-J.** Rescue experiments. CCK8, colony formation, and flow cytometry assays were conducted on cells in four treatment groups (si-NC + vector, si-NC + pcDNA CIRBP, si-circ_0007386 + vector, si-circ_0007386 + pcDNA CIRBP). Data are shown as means ± SD. **p* < 0.05, ***p* < 0.01, ****p* < 0.001, *****p* < 0.0001.


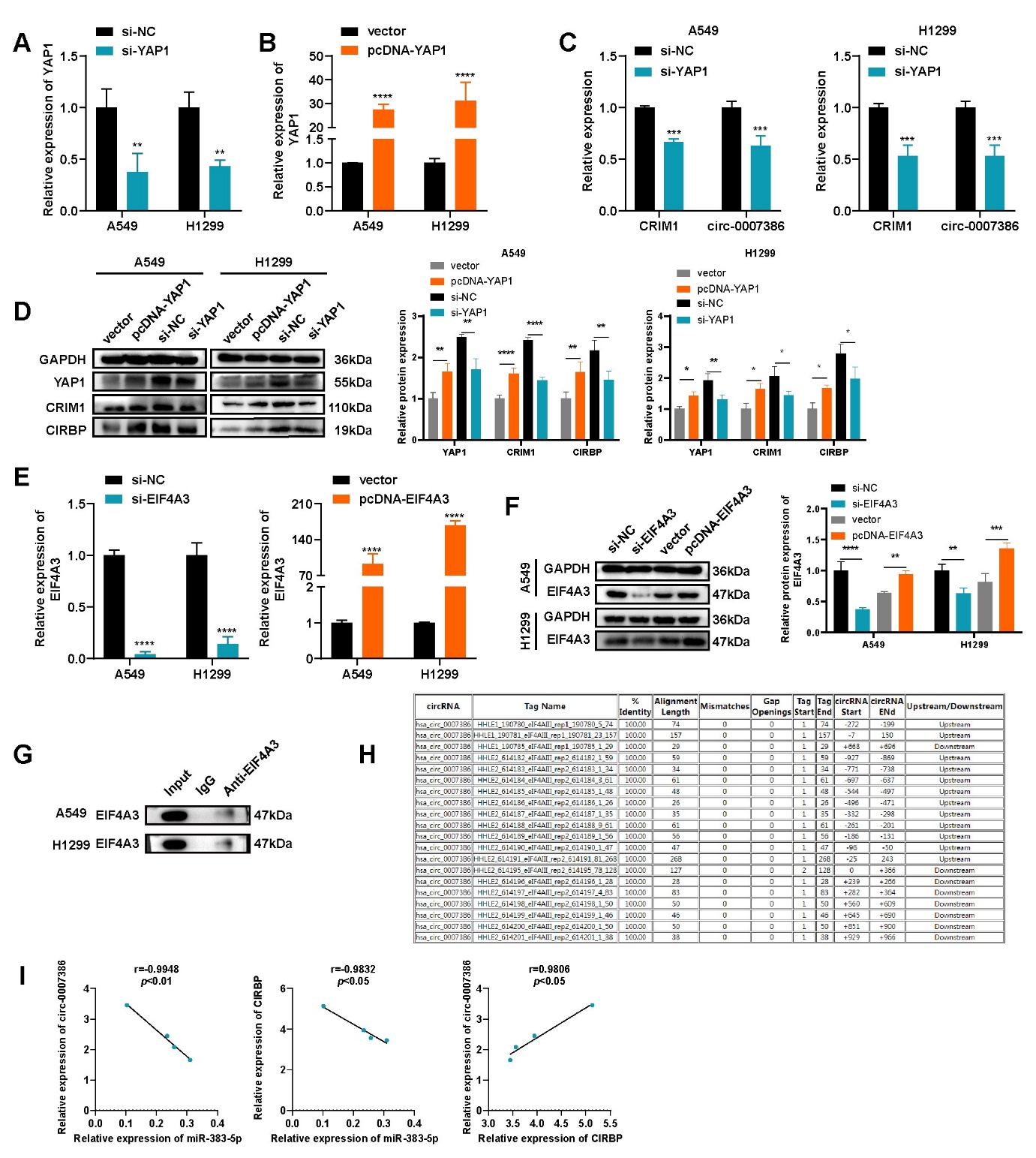


**Fig. S4 YAP1 and EIF4A3 regulate the expression of circ_0007386. A-B.** Relative expression of *YAP1* mRNA in A549 and H1299 cells transfected with the short hairpin RNA of YAP1 (si-YAP1) and an overexpression vector of YAP1 (pcDNA-YAP1) determined by RT-qPCR. **C.** The relative expression of *CRIM1* mRNA and circ_0007386 after YAP1 knockdown was detected by RT-qPCR. **D.** The relative expression of YAP1, CRIM1, and CIRBP in A549 and H1299 cells transfected with the indicated vectors or siRNAs. **E-F.** Relative expression of EIF4A3 mRNA and protein in A549 and H1299 cells transfected with the short hairpin RNA of EIF4A3 (si-EIF4A3) and an overexpression vector of EIF4A3 (pcDNA-EIF4A3) . **G.** RIP assays using a EIF4A3 antibody were performed to examine interactions with *CRIM1* pre-mRNA transcript in the indicated groups. **H.** EIF4A3 binding sites in introns flanking the circularized exons in *CRIM1* pre-mRNA predicted by CircInteractome. Data are shown as means ± SD. **I**. Correlation analysis of circ_0007386, miR-383-5p, and CIRBP expression analyzed using RT-qPCR in NSCLC cells. **p* < 0.05, ***p* < 0.01, ****p* < 0.001, *****p* < 0.0001.


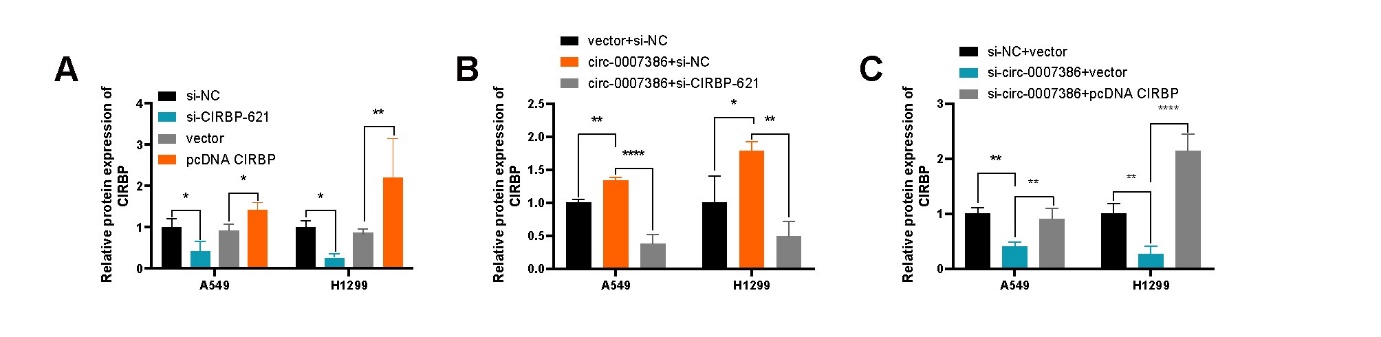


**Fig. S5 The quantitative analysis of western blot data of Fig. 5I-J. A.** Quantitative analysis of CIRBP protein levels in cells transfected with miR-383-5p mimics and inhibitor and its controls. **B-C.** Quantitative analysis of CIRBP protein levels in the indicated groups (si-NC + NC-inhibitor, si-circ_0007386 + NC-inhibitor, si-circ_0007386 + miR-383-5p inhibitor and vector + mimics NC, circ_0007386 + NC-mimics, circ_0007386 + miR-383-5p mimics). Data are shown as means ± SD. **p* < 0.05, ***p* < 0.01, ****p* < 0.001, *****p* < 0.0001.


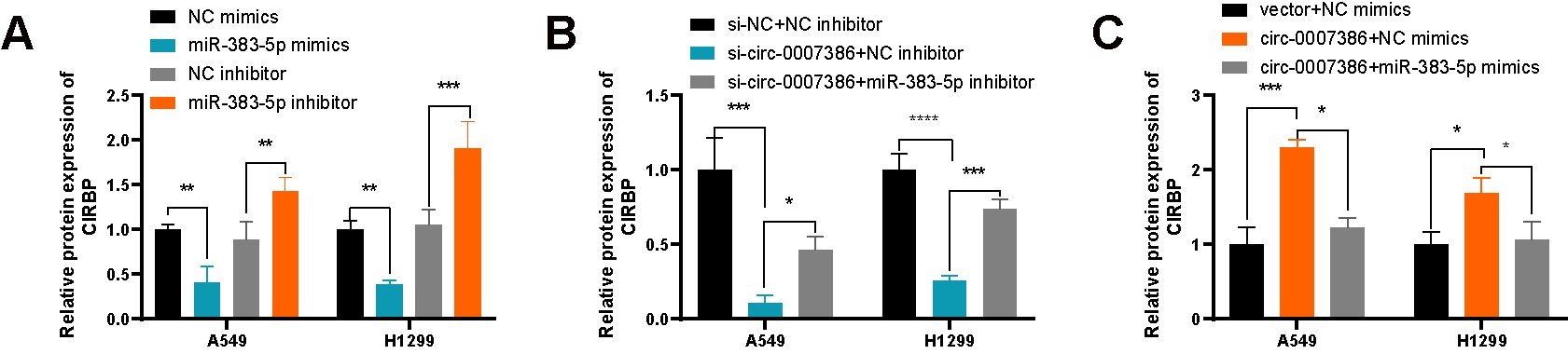


**Fig. S6 The quantitative analysis of western blot data of Fig. 6C-E. A.** Quantitative analysis of CIRBP protein levels in cells transfected with si-CIRBP and pcDNA CIRBP and its controls. **B-C.** Quantitative analysis of CIRBP protein in cells across indicated groups (vector + si-NC, circ_0007386 + si-NC, circ_0007386 + si-CIRBP-621 and si-NC + vector, si-circ_0007386 + vector, si-circ_0007386 + pcDNA CIRBP). Data are shown as means ± SD. **p* < 0.05, ***p* < 0.01, ****p* < 0.001, *****p* < 0.0001.


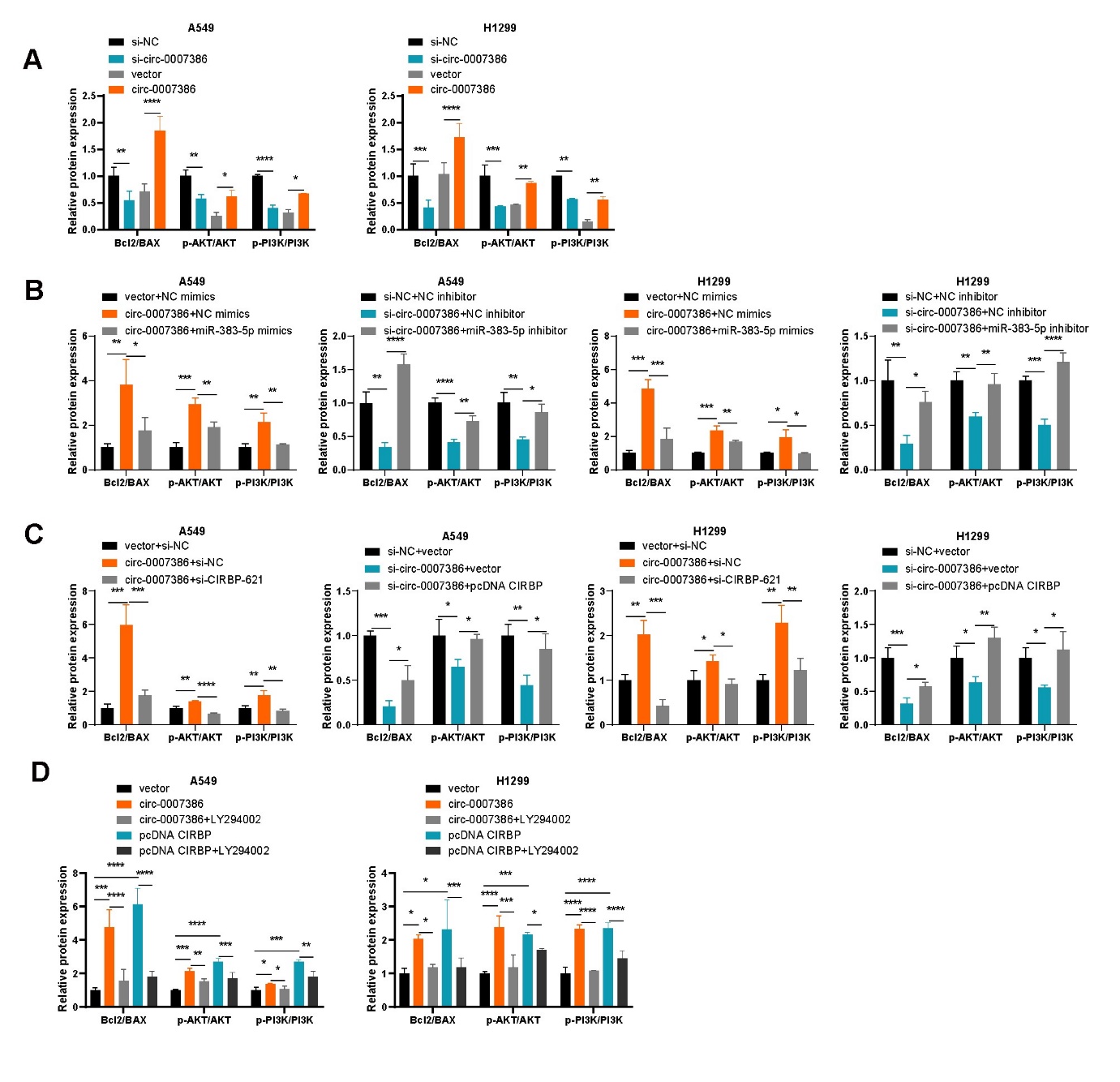


**Fig. S7 The quantitative analysis of western blot data of Fig. 7A-D. A.** Quantitative analysis of p-AKT/AKT, p-PI3K/PI3K, and apoptosis-associated markers in cells with circ_0007386 knockdown or overexpression treatment. **B-C.** Quantitative analysis of p-AKT/AKT, p-PI3K/PI3K, and apoptosis-associated markers in cells treated as indicated. **D.** Quantitative analysis of p-AKT/AKT, p-PI3K/PI3K, and apoptosis-associated markers in cells transfected with the indicated vectors and treated with the PI3K/AKT pathway inhibitor (LY294002). Data are shown as means ± SD. **p* < 0.05, ***p* < 0.01, ****p* < 0.001, *****p* < 0.0001.
