# Supplementary material for: Hypoxia-enhanced YAP1-EIF4A3 interaction drives circ_0007386 circularization by competing with CRIM1 pre-mRNA linear splicing and promotes non-small cell lung cancer progression
Source: J Exp Clin Cancer Res. 2024 Jul 20;43:200. doi: 10.1186/s13046-024-03116-6 (PMC11264895; doi:10.1186/s13046-024-03116-6)
Supplement: Supplementary file 2 — Supplementary Material 2 [file 13046_2024_3116_MOESM2_ESM.pdf]

Table S1 Probes and siRNAs used in the experiments.

名称	序列(5′ → 3′)
Negative control	UUCUCCGAACGUGUCACGUTT
	ACGUGACACGUUCGGAGAATT
si-has_circ_0007386	CCAACAAGAAGAGAAGCCATT
	UGGCUUCUCUUCUUGUUGGTT
miR-383-5p mimics	AGAUCAGAAGGUGAUUGUGGCU
	CCACAAUCACCUUCUGAUCUUU
inhibitor NC	CAGUACUUUUGUGUAGUACAA
miR-383-5p inhibitor	AGCCACAAUCACCUUCUGAUCU
si-CIRBP-242	GAUCUCUGAAGUGGUGGUUTT
	AACCACCACUUCAGAGAUCTT
si-CIRBP-344	GGCCAUGAAUGGGAAGUCUTT
	AGACUUCCCAUUCAUGGCCTT
si-CIRBP-621	GGGUCCUACAGAGACAGUUTT
	AACUGUCUCUGUAGGACCCTT
si-YAP1-homo	GGUGAUACUAUCAACCAAATT
	UUUGGUUGAUAGUAUCACCTT
si-EIF4A3-homo	UCAUAUCAAAAACACGCCCTT
	GGGCGUGUUUUUGAUAUGAUU
