# Supplementary material for: Hypoxia-enhanced YAP1-EIF4A3 interaction drives circ_0007386 circularization by competing with CRIM1 pre-mRNA linear splicing and promotes non-small cell lung cancer progression
Source: J Exp Clin Cancer Res. 2024 Jul 20;43:200. doi: 10.1186/s13046-024-03116-6 (PMC11264895; doi:10.1186/s13046-024-03116-6)
Supplement: Supplementary file 3 — Supplementary Material 3 [file 13046_2024_3116_MOESM3_ESM.pdf]

Table S2 Primers used in the experiments.

名称	序列(5′ → 3′)
Hsa circ:chr3:169854206-169867032	F:CAAGCCTGGAATCACGAAGC
	R:GTTGGGTAATACTGCCGCTG
Hsa circ:chr9:138709822-138774924	F: AAGTCAAAGCCGAAGAAGCC
	R: CCTGCTCATACTGGTCAACG
Hsa circ:chr4:187560875-187584767	F: ACGTCAAGGAAGCTAGACCG
	R: TGAACGATGAAAGAGGCACG
Hsa circ:chr9:96233422-96261168	F:ACCACATTACTTAGGTTGC AC AG
	R:CGTTCCGCCTCAGTTTTAGG
Hsa circ:chr5:138614015-138614818	F: TGAATGACATCTACCTCCATCAG
	R: ACCTGCCACTATTTCCTCCC
Has_circ_0007386	F: GCTGTGACCTCTATGAGTGC
	R: GGCTTCTCTTCTTGTTGGCA
Has_circ_0007386(convergent)	F:AGCTTGACTTCTGGCCTTCT
	TCTCTGACCTTCTCTGGGGA
β-actin(h)	F: AGCGAGCATCCCCCAAAGTT
	R: GGGCACGAAGGCTCATCATT
U6	F: CTCGCTTCGGCAGCACA
	R: AACGCTTCACGAATTTGCGT
Hsa-miR-383-5p	R: AGATCAGAAGGTGATTGTGGCT
CIRBP	F: TGCCCCACTCACTTCTCT
	R: CGGGGTCCTACCACACT
CRIM1	F: CAGAACAAGCATTCCCACT
	R: CCATCCAAACTCACTGTCC
EIF4A3	F: AAGGGAGAGATGTCATCGCAC
	R: GCTTGAGTTTCACGAACCTGA
YAP1	F: TAGCCCTGCGTAGCCAGTTA
	R: TCATGCTTAGTCCACTGTCTGT
CRIM1 pre-mRNA	F: ATGCCCATCCATTCTCACCA
	R: GGTGGTGGAGGATGACAGAA
