# Supplementary material for: Hypoxia-enhanced YAP1-EIF4A3 interaction drives circ_0007386 circularization by competing with CRIM1 pre-mRNA linear splicing and promotes non-small cell lung cancer progression
Source: J Exp Clin Cancer Res. 2024 Jul 20;43:200. doi: 10.1186/s13046-024-03116-6 (PMC11264895; doi:10.1186/s13046-024-03116-6)
Supplement: Supplementary file 4 — Supplementary Material 4 [file 13046_2024_3116_MOESM4_ESM.pdf]

Table S3.Antibodies used in the present study.

Product	Source	No. of Catalogue
**Western blot:**		
GAPDH	Bioworld	AP0066
Anti-β-Actin	Cell Signaling Technology	4970S
Anti-CIRBP	Proteintech	10209-2-AP
Anti-Bcl2	Beyotime	AF6285
Anti-BAX	Beyotime	50599-2-Ig
Anti-Phospho-PI3K	Beyotime	AF5905
Anti-PI3K	Beyotime	AF7742
Anti-AKT	Beyotime	AA326
Anti-Phospho-AKT1	Beyotime	AF5740
Anti-YAP1	Bioss	bs-3605R
Anti-CRIM1	Bioss	bs-2034R
Anti-EIF4A3	Proteintech	17504-1-AP
**Secondary antibody**		
Anti-Rabbit IgG(H+L)	Proteintech	SA00001-2
**IHC**		
Anti-Ki67	servicebio	GB111499
Anti-CIRBP	Proteintech	10209-2-AP
Anti-BAX	Proteintech	50599-2-Ig
Anti-Bcl2	servicebio	GB113375
**RIP**		
IgG	Merck	PP64B
Anti-EIF4A3	Proteintech	17504-1-AP
**CoIP**		
IgG	Cell Signaling Technology	3900S
Anti-EIF4A3	Proteintech	17504-1-AP
Anti-YAP1	Bioss	bs-3605R
