# Supplementary material for: Hypoxia-enhanced YAP1-EIF4A3 interaction drives circ_0007386 circularization by competing with CRIM1 pre-mRNA linear splicing and promotes non-small cell lung cancer progression
Source: J Exp Clin Cancer Res. 2024 Jul 20;43:200. doi: 10.1186/s13046-024-03116-6 (PMC11264895; doi:10.1186/s13046-024-03116-6)
Supplement: Supplementary file 5 — Supplementary Material 5 [file 13046_2024_3116_MOESM5_ESM.pdf]

**Table S4** Candidate miRNAs binding to circ_0007386 predicted by CircInteractome.

<table>
<tr><th>CircRNA<br>Mirbase ID</th><th>CircRNA (Top) - miRNA (Bottom) pairing</th><th>Site Type</th></tr>
<tr><td>hsa_circ_0007386 (5' ... 3')<br>hsa-miR-1184 (3' ... 5')</td><td>CUCAAGUCAGACUAACUGCAGAU<br>||| ||||||<br>CCUUCGGUAGUUCAGC-GACGUCC</td><td>7mer-1a</td></tr>
<tr><td>hsa_circ_0007386 (5' ... 3')<br>hsa-miR-1205 (3' ... 5')</td><td>CUCAAGUCAGACUAACUGCAGAU<br>||||| ||||||<br>GAGUUUCGUUUGG--GACGUCU</td><td>7mer-1a</td></tr>
<tr><td>hsa_circ_0007386 (5' ... 3')<br>hsa-miR-1324 (3' ... 5')</td><td>GUGCAACCCCGCAGGCUGUCUGC<br>|||||||<br>CUUUCACGUAUCUUAAGACAGACC</td><td>7mer-m8</td></tr>
<tr><td>hsa_circ_0007386 (5' ... 3')<br>hsa-miR-149 (3' ... 5')</td><td>NNNNNNNNNNNAAGAGA---AGCCAGAU<br>|||| ||||||<br>CCCUCACUUCUGUGCCUCGGUCU</td><td>7mer-1a</td></tr>
<tr><td>hsa_circ_0007386 (5' ... 3')<br>hsa-miR-182 (3' ... 5')</td><td>GAUGGUUGCUGUACUUUGCCAAC<br>|||||||<br>UCACACUCAAGAUGGUAACGGUUU</td><td>7mer-m8</td></tr>
<tr><td>hsa_circ_0007386 (5' ... 3')<br>hsa-miR-383 (3' ... 5')</td><td>UCCUGAAGAUUCUGUUCUGAUCG<br>|||||||<br>UCGGUGUUAGUGGAAGACUAGA</td><td>7mer-m8</td></tr>
<tr><td>hsa_circ_0007386 (5' ... 3')<br>hsa-miR-665 (3' ... 5')</td><td>UCGAGGGUUAUGCUCCUCCUGGG<br>|||||||<br>UCCCCGGAGUCGGAGGACCA</td><td>7mer-m8</td></tr>
<tr><td>hsa_circ_0007386 (5' ... 3')<br>hsa-miR-942 (3' ... 5')</td><td>NNNNNNNNNNNNNNNNAAGAGAAGC<br>|||||||<br>GUGUACCGGUUUUGUCUCUUCU</td><td>7mer-m8</td></tr>
<tr><td>hsa_circ_0007386 (5' ... 3')<br>hsa-miR-432 (3' ... 5')</td><td>AGAGAAGCCAGAUUGCUCCAAGG<br>|||||||<br>GGUGGGUUACUGGAUGAGGUUCU</td><td>7mer-m8</td></tr>
<tr><td>hsa_circ_0007386 (5' ... 3')<br>hsa-miR-507 (3' ... 5')</td><td>CUGUGACCUCUAUGAGUGCAAAC<br>|||||||<br>AAGUGAGGUUUUCCACGUUUU</td><td>7mer-m8</td></tr>
<tr><td>hsa_circ_0007386 (5' ... 3')<br>hsa-miR-557 (3' ... 5')</td><td>CUGUGACCUCUAUGAGUGCAAAC<br>|||||||<br>UCUGUUCCGGGUGGGCACGUUUG</td><td>7mer-m8</td></tr>
<tr><td>hsa_circ_0007386 (5' ... 3')<br>hsa-miR-513a-5p (3' ... 5')</td><td>UUGCUCCAAGGCCCGCUGUGAAG<br>||||||<br>UACUGUGGAGGGACACUU</td><td>7mer-1a</td></tr>
<tr><td>hsa_circ_0007386 (5' ... 3')<br>hsa-miR-548k (3' ... 5')</td><td>CUGCAGAUGGUUGCUGUACUUUG<br>|||||||<br>UCGUUUUAGGCGUUCAUGAAAA</td><td>7mer-m8</td></tr>
<tr><td>hsa_circ_0007386 (5' ... 3')<br>hsa-miR-555 (3' ... 5')</td><td>UGGGGAGUGCUGUCCCUUACCCA<br>|||||||<br>UAGUCUCCAAGUCGAAUGGGA</td><td>8mer-1a</td></tr>
<tr><td>hsa_circ_0007386 (5' ... 3')<br>hsa-miR-567 (3' ... 5')</td><td>CAGCCGGGAAACCUG----AACAUACU<br>|||| |||||||<br>CAAGACAGGACCUUCUUGUAUGA</td><td>7mer-m8</td></tr>
</table>
